# Transcript Engineered Extracellular Vesicles Alleviate Alloreactive Dynamics in Renal Transplantation

**DOI:** 10.1002/advs.202202633

**Published:** 2022-09-08

**Authors:** Jinwen Lin, Junhao Lv, Shiping Yu, Ying Chen, Huiping Wang, Jianghua Chen

**Affiliations:** ^1^ Kidney Disease Center The First Affiliated Hospital Zhejiang University School of Medicine Key Laboratory of Kidney Disease Prevention and Control Technology National Key Clinical Department of Kidney Diseases Institute of Nephrology Zhejiang University, and Zhejiang Clinical Research Center of Kidney and Urinary System Disease Hangzhou Zhejiang Province 310003 P. R. China; ^2^ Zhejiang University‐University of Edinburgh Institute School of Medicine Zhejiang University Hangzhou Zhejiang Province 310003 P. R. China

**Keywords:** allograft rejection, genetic engineering, immune adhesion, renal transplantation

## Abstract

Direct contact of membrane molecules and cytokine interactions orchestrate immune homeostasis. However, overcoming the threshold of distance and velocity barriers, and achieving adhesion mediated immune interaction remain difficult. Here, inspired by the natural chemotaxis of regulatory T cells, multifunctionalized FOXP3 genetic engineered extracellular vesicles, termed Foe‐TEVs, are designed, which display with adhesive molecules, regulatory cytokines, and coinhibitory contact molecules involving CTLA‐4 and PD‐1, by limited exogenous gene transduction. Foe‐TEVs effectively adhere to the tubular, endothelial, and glomerular regions of allogeneic injury in the renal allograft, mitigating cell death in situ and chronic fibrosis transition. Remarkably, transcript engineering reverses the tracking velocity of vesicles to a retained phenotype and enhanced arrest coefficient by a factor of 2.16, directly interacting and attenuating excessive allosensitization kinetics in adaptive lymphoid organs. In murine allogeneic transplantation, immune adhesive Foe‐TEVs alleviate pathological responses, restore renal function with well ordered ultrastructure and improved glomerular filtration rate, and prolong the survival period of the recipient from 30.16 to 92.81 days, demonstrating that the delivery of extracellular vesicles, genetically engineered for immune adhesive, is a promising strategy for the treatment of graft rejection.

## Graphical abstract

Inspired by the natural chemotaxis of regulatory T cells to immune effector regions, FOXP3 gene engineering extracellular vesicles are designed with adhesive molecules, anti‐inflammatory cytokines as well as coinhibitory contact molecules by limited exogenous gene transduction to carry contact and secretory immune modulation.

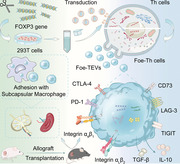

## 1 Introduction

Effective adhesion to immune effector cells or regions is key to ensure the close‐range interaction of cytokines and realize immune regulation.^[^ ^1^ ^]^ Despite therapeutic regimens, involving recombinant interleukin 10 (IL‐10), transforming growth factor *β* (TGF‐*β*), as well as monoclonal antibodies, secretory cytokines known as paracrine communicate over relatively short distances, depending on the proximity and retention in space.^[^ ^2^ ^]^ Meanwhile, the systemic administration of these biologics may be insufficient to achieve adequate concentration at the target sites for close‐range interaction, particularly with short half‐life and duration in vivo. Importantly, due to powerful and rapid processing and clearance of abundant microcirculation drainage fluids, it is difficult to realize effectively adhesion and arresting by flow‐induced adaptive immune in secondary lymphoid tissue, further impeding the interactions of secretory cytokines.^[^ ^3^ ^]^

Direct contact formed by immune adhesion provides essential instructional signals for immune regulation, which cannot be readily achieved using soluble agents that exhibit transient effects.^[^ ^4^ ^]^ Through adherence to the effector cell, a specific contact region of immunological synapse formed, with the engagement of accessory receptors and immune‐related molecules such as CTLA‐4 and PD‐1 to trigger surface receptors.^[^ ^5^ ^]^ Recently, inspired by regulatory T (Treg) cells regulating the effectors through surface coinhibitory contact, the immunosuppression of immune synapse construction is simulated to a certain extent by the infusion of natural Treg cells.^[^ ^6^ ^]^ However, the immunomodulatory effect reported in clinical studies is not satisfactory due to poor engraftment and cell survival of the infused cells.^[^ ^7^ ^]^ Meanwhile, phenotype drift and continuous transcription of target genes in orchestrating immune homeostasis have also been major obstacles.^[^ ^8^ ^]^ Accumulating evidence identified the extracellular vesicles (EVs) derived from Treg cells as a novel immune regulatory method.^[^ ^9^ ^]^ However, using naive Treg cell‐derived EVs directly as therapeutic method are restricted by the scarcity of Treg cells, which constitute only 5–10% of all the circulating CD4^+^ T cells.^[^ ^10^ ^]^ Additionally, the nondeterminacy about the regulatory and proliferative potential in the in vitro expansion also raise concerns for the large‐scale manufacturing Treg cell‐derived EVs.^[^ ^11^ ^]^ To date, the identification of alternative Treg cells derivatives that elicit not only Treg cell contact‐dependent suppression but also soluble factors secretion remains difficult.

The kidney is the most commonly replaced organ, and renal allografts are prone to immune rejection. Once the allograft is transplanted in recipient, a large number of alloantigens quickly drain into lymphoid organs to activate adaptive response, and form more serious graft injury and antigen abscission.^[^ ^12^ ^]^ Therefore, alternative strategies to concurrently solve the aforementioned problems, including the prevention of immune allorecognition, attenuated allosensitization kinetics in secondary lymphoid organs, restoration of renal architecture and glomerular filtration, present a blueprint for synergetic treatment of allograft rejection. EVs carry the cargoes of mRNAs, microRNAs, cytokines as well as membrane molecules, mediating signal transduction among cells.^[^ ^13^ ^]^ Importantly, recent researches have demonstrated the preserving effects of EVs encapsulation compared to free cytokines. Furthermore, nucleic acids and proteins show high stability inside EVs, which directly overcome the limits of systemic administration of cytokines.^[^ ^14^ ^]^ Currently, genetically engineered EVs with enhanced bioactivity, stability or targeting have emerged as a versatile platform in various pathologies.^[^ ^14^, ^15^ ^]^ However, integration and insertion of the foreign gene need genome cleaving. It should be point out that unexpected insertions or deletions during gene disruption may cause a functional loss of the host protein or even cell death, especially in eukaryotes.^[^ ^16^ ^]^ Meanwhile, multi gene transfection requires long cloning cycle and demonstrated low success rate, thus engineering strategies can only realize one or a few exogenous gene transductions, contributing to restricted targeted gene products.^[^ ^17^ ^]^ To date, engineered EVs capable of both contacting and secretory dependent immunosuppressive approaches with limited exogenous genes transduction have not been reported.

**Scheme 1:**
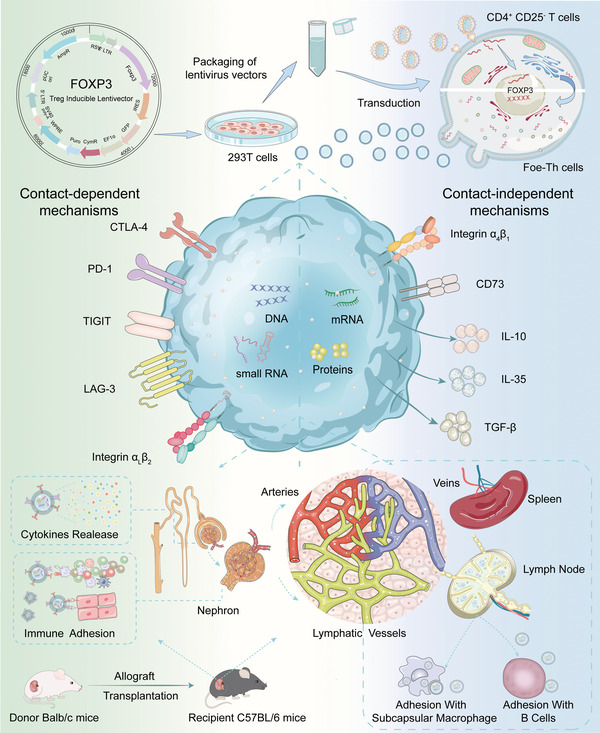
Schematic illustration of the immune adhesive FOXP3 genetic engineering extracellular vesicles alleviates renal allograft rejection via contact dependent and secretory cytokines pathways.

To address these challenges, we have constructed multifunctionalized immune adhesive FOXP3 transcriptional gene engineered extracellular vesicles, termed Foe‐TEVs, which present with adhesive molecules, anti‐inflammatory cytokines as well as coinhibitory contact molecules involving CTLA‐4 and PD‐1, by limited exogenous gene transduction (**Scheme** **1**). With abundant adhesive molecules of integrins, Foe‐TEVs localized to the tubular epithelial cells (TECs), endothelial cells, and glomerular regions of allogeneic immune injury in the renal allograft maintaining retention for more than 192 h, alleviating allograft‐targeting immune responses, and mitigating apoptosis in situ and chronic fibrosis transition. Particularly, the mean velocity of the adhesive Foe‐TEVs was significantly lower by 20%, while with a higher arrest coefficient by 2.16 times compared with Vector‐TEVs in secondary lymph organs. As a result, it was directly exhibited in suppressing the biased polarization of Th1 cells with interferon‐g (IFN‐g) decreased by 76.19%, inhibiting B cells alloimmunity with depressed donor specific antibody (DSA) production and hindering complement activation. In murine allogeneic transplantation, immune adhesive Foe‐TEVs alleviated graft pathological lesions including arteritis, tubulitis, interstitial fibrosis, and so on, restored renal function with well‐ordered ultrastructure, improved glomerular filtration and prolonged the survival period of the recipient from 30.16 to 92.81 days. Collectively, through the identification and loading of broad‐spectrum adhesion molecules, the extracellular vesicles, engineered edited by selected transcript, realized the regulation of alloreactive responses via the direct contact and the close‐range interaction of cytokines, providing a promising strategy for the treatment of graft rejection.

## 2 Results and Discussion

### 2.1 Preparation and Characterization of the Foe‐TEVs

**Figure 1:**
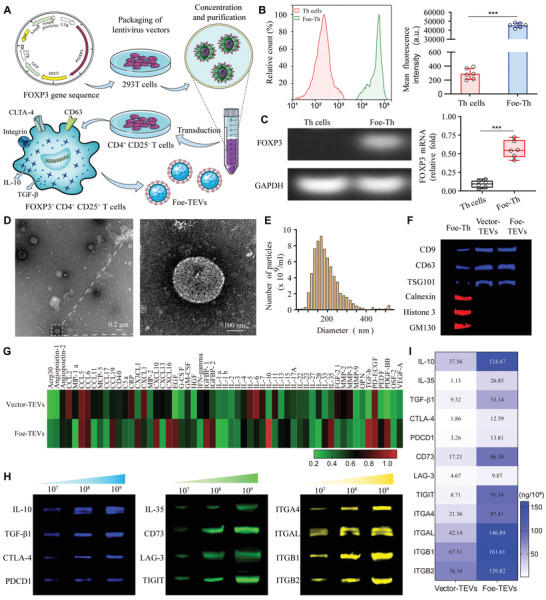
Establishment and characterization of Foe‐TEVs. A) The schematic illustration of Foe‐TEVs establishment. B) Mean fluorescence intensity and relative count of the reporter in Th cells and Foe‐Th. C) The mRNA level of FOXP3 relative with GAPDH analyzed by northern blot in Th cells and Foe‐Th. D) Transmission electron microscopy imaging of Foe‐TEVs. E) Nanoparticle tracking analysis of Foe‐TEVs. F) Western blot analysis of CD9, CD63, TSG101, Calnexin, Histone 3, and GM130 expression in Foe‐Th cells, Vector‐TEVs, and Foe‐TEVs. G) Proteomic characterization of cytokines in Vector‐TEVs and Foe‐TEVs. H) Western blotting analysis of the expression of IL‐10, TGF‐*β*1, CTLA‐4, PDCD1, CD35, CD73, LAG3, TIGIT, and subunits of integrins including ITGA4, ITGAL, ITGB1, and ITGB2 on Foe‐TEVs with different concentrations. I) Analysis of various protein contents in Vector‐TEVs and Foe‐TEVs at 1 × 10^9^ particles. ***P < 0.001; Values are presented as mean ± SEM, *n* = 6. Statistical analysis was determined by two‐tailed Student's *t*‐test.

FOXP3 sequence was first transfected into 293T cells using Treg cell inducible lentivector to construct lentivirus vectors, as shown in **Figure** **1**A. Then, lentiviral vectors containing the FOXP3 gene were introduced into CD4^+^ Th cells isolated from the mice spleens with 95.23% purity to genetically edit T cells, while in the control group only the vector was transfected into CD4^+^ Th cells (Figure S1, Supporting Information). In order to verify the successful transfection of CD4^+^CD25^−^ T cells (Th cells), flow cytometry was conducted to detect reporter expression and observed as high as 5 × 10^4^ of GFP mean fluorescence intensity in FOXP3 engineered CD4^+^CD25^−^ T cells (Foe‐Th) (Figure 1B). Furthermore, northern blot of mRNA of FOXP3 verified that lentiviral vectors resulted in an increase of FOXP3 relative expression from 0.11 ± 0.01 to 0.56 ± 0.04 in Foe‐Th compared to Th cells (Figure 1C), indicating that the transcripts were stably expressed and formed functional mRNA. Afterward, Foe‐TEVs isolated from Foe‐Th cells were prepared and purified by differential centrifugation, and verified the presence of EVs on the basis of morphology, size, and protein markers. First, the presence of Foe‐TEVs was confirmed by transmission electron microscopy (TEM), with a regular spherical shape and the lipid bilayer structure (Figure 1D). Nanoparticle tracking analysis (NTA) revealed that the diameter of the Foe‐TEVs ranged from 50 to 500 nm, with a mean diameter of 196.34 ± 8.63 nm (Figure 1E). Also, the TEM and NTA results of Vector‐TEVs have been shown in Figure S2 of the Supporting Information suggesting no difference from Foe‐TEV. Western blot analysis revealed the enrichment of EVs associated markers including CD9 CD63 and TSG101 in Vector‐TEVs and Foe‐TEVs compared with Foe‐Th. By contrast, the negative markers on EVs including calnexin, histone 3, and GM13 were highly expressed in Foe‐Th cells, but hardly detection in isolated Vector‐TEVs and Foe‐TEVs (Figure 1F). Thus, the above results supported the relative purity of the isolated vesicles. Next, high throughput proteomics were performed to characterize the content of cytokines in the engineered vesicles. It was shown that regarding the spectrum of inhibitory immune factors, multiple cytokines such as IL‐10, TGF‐*β*1, and PDGF‐BB of Foe‐TEVs were significantly upregulated compared to those of Vector‐TEVs, whereas some typical proinflammatory factors were reduced, providing a basis for effective immune regulation (Figure 1G). Meanwhile, no obvious difference has been observed between the Foe‐TEVs and the natural Treg‐EVs, from measurements of transcriptional level of corresponding source cells, markers, and diameter distribution as well as the protein composition in EVs (Figure S3, Supporting Information). Moreover, with the increasing concentration of Foe‐TEVs, the capacity of suppressive cytokines was increased gradually. Notably, we found that the contact immunomodulatory molecules including CTLA‐4, TIGIT, LAG‐3, and CD73 as well as secretary immunomodulation cytokines involving IL‐10, IL‐35, and TGF‐*β* were effectively carried by the Foe‐TEVs, and these protein contents increased with Foe‐TEVs dose. Meanwhile, with the increase of the EVs dose, the integrin subunit contents of ITGA4, ITGAL, ITGB1, and ITGB2 in Foe‐TEVs also elevated (Figure 1H). Consistently, with a variety of immune regulatory molecules, Foe‐TEVs demonstrated distinct immunosuppressive effects toward T cell polarization stimulated by allograft lysate. With increasing dosage of Foe‐TEVs, the inhibitory effect of alloreactive T cell response increased until 1 × 10^9^ particles, indicating the appropriate Foe‐TEVs dose to be used in the subsequent therapeutic trial (Figure S4, Supporting Information). Additionally, the level of functional proteins at 1 × 10^9^ particles of Vector‐TEVs and Foe‐TEVs were also characterized by enzyme‐linked immunosorbent assay (ELISA) assay shown in Figure 1I. It was revealed that Foe‐TEVs at this dose expressed higher levels of immunoregulation proteins with 12.59 ± 0.94 ng of CTLA‐4, 91.54 ± 10.36 ng of TIGIT, and 53.14 ± 4.68 ng of TGF‐*β*1. Meanwhile, the results also showed that Foe‐TEVs at 1 × 10^9^ particles contained IL‐10 as high as 124.47 ng and displayed abundant integrin contents. To determine the contribution of coisolated components to therapeutic effects, cytokines quantification of lysed and no lysed EVs was performed. Although, a trace of cytokines was detected in the no lysed EVs, which may be originated from the coisolation of soluble proteins and was consistent with previous findings.^[^ ^18^ ^]^ The total proteins level in the lysed EVs was higher than that of the no lysed EVs, with a mean of 139.71 versus 1268.94 ng per 10^9^ EVs. Specifically, the cytokines level of IL‐10, TGF‐*β*1, and IL‐35 in lysed EVs were more than ten times as the no lysed group, indicating the EVs constitute the main contribution to the therapeutic efficacy (Figure S5, Supporting Information). Collectively, Foe‐TEVs genetically engineered by selected exogenous transcript, achieved stable expression of the FOXP3 transcription factor and its target gene products.

### 2.2 Evaluation of In Vitro Biocompatibility

**Figure 2:**
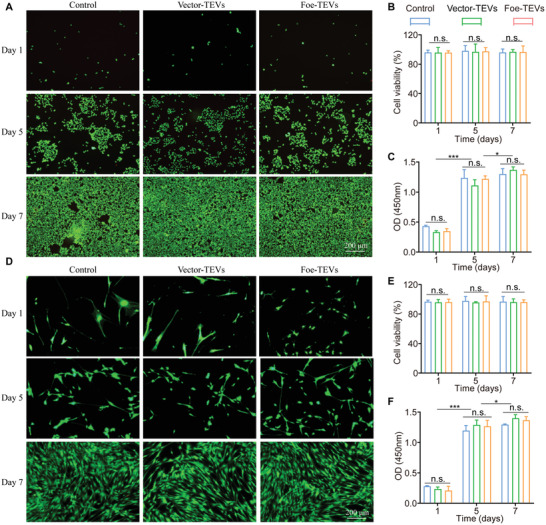
Biocompatibility evaluation of Foe‐TEVs in vitro. A) Live/dead fluorescence results of Vector‐TEVs and Foe‐TEVs cocultured with HUVECs at day 1, 5, and 7, respectively. B) Cell viability of control, Vector‐TEVs, and Foe‐TEVs cocultured with HUVECs at day 1, 5, and 7, respectively. C) CCK‐8 quantification results in control, Vector‐TEVs, and Foe‐TEVs cocultured with HUVECs at day 1, 5, and 7, respectively. D) Live/dead fluorescence results of Vector‐TEVs and Foe‐TEVs cocultured with KFBs at day 1, 5, and 7, respectively. E) Cell viability of control, Vector‐TEVs, and Foe‐TEVs cocultured with KFBs at day 1, 5, and 7, respectively. F) CCK‐8 quantification results in control, Vector‐TEVs, and Foe‐TEVs cocultured with KFBs at day 1, 5, and 7, respectively. HUVECs, human umbilical vein endothelial cells; KFBs, kidney fibroblasts. n.s., not significant; *P < 0.05, ***P < 0.001; Values are presented as mean ± SEM, *n* = 6. Statistical analysis was performed by the one‐way analysis of variance.

To ascertain the safety and effectiveness of Foe‐TEVs, biocompatibility studies were carried out in vitro by incubating Foe‐TEVs or Vector‐TEVs with human umbilical vein endothelial cells (HUVECs) and kidney fibroblasts (KFBs) for 7 days. It was shown that HUVECs and KFBs in all three groups could survive and proliferate at a comparative level to an appropriate control, indicating the safety of Foe‐TEVs at concentrations in a wide range of 10^7^–10^11^ particles (Figures S6 and S7, Supporting Information). Assessing cell viability with live/dead staining, the results showed that cells in three groups could survive and proliferate well, with less than 5% mortality (**Figure** **2**A,B,D,E). It was noted that the quantitative statistics further supported the excellent biocompatibility of Foe‐TEVs group compared with control group (Figure 2B,E). Proliferative cytotoxicity is an important parameter for evaluating the clinical application in the stage of preclinical experiment.^[^ ^19^ ^]^ Subsequently, CCK‐8 assay was performed to better understand whether Foe‐TEVs could influence cell proliferation. Cells in all three groups were cultured with similar growth trend over time, and there was no statistical difference in proliferation at the same day among the control, Vector‐TEVs, and Foe‐TEVs groups (Figure 2C,F). Furthermore, in vivo experiments also revealed that Foe‐TEVs possessed great biocompatibility. The results showed that the body weights, blood cell counts, representative inflammatory factor levels, liver function as well as renal function of mice in Foe‐TEVs group were not significantly different from those of healthy mice on day 7 and day 28 (Figures S8–S11, Supporting Information).

### 2.3 Foe‐TEVs Targeted into the Allograft, Allograft Draining Lymph Nodes (ADLNs), and Spleen Mediated by Immune Adhesion

**Figure 3:**
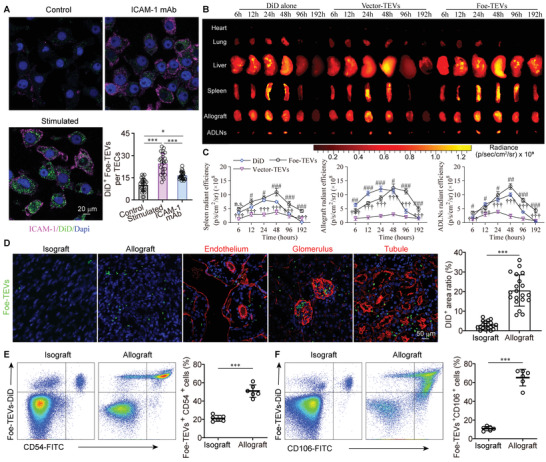
Foe‐TEVs targeted into the allograft, ADLNs, and spleen mediated by immune adhesion. A) Confocal imaging and statistical data of DiD‐labeled Foe‐TEVs adhere to ICAM‐1 on HK‐2 cells in control group, stimulated group, and VCAM‐1 monoclonal antibody blocking group (*n* = 25). *P < 0.05, ***P < 0.001. B) Radiance changes and distribution of DiD alone, Vector‐TEVs, and Foe‐TEVs in indicated organs at 6, 12, 24, 48, 96, and 192 h after injection. C) Quantitative radiant efficiency in spleen, ADLNs, and renal allograft at different time series (*n* = 3). ^#^ *P* < 0.05, ^##^ *P* < 0.01, and ^###^ *P* < 0.001 compared to the DiD group; ^†††^ *P* < 0.001 compared to the Vector‐TEVs group; n.s., no significance. D) The distribution of Foe‐TEVs in both isograft and allograft groups detected by immunofluorescence (*n* = 20). E) Quantification by flow cytometry of Foe‐TEVs^+^ CD54^+^ cells in different groups (*n* = 6). F) Quantification by flow cytometry of Foe‐TEVs^+^ CD106^+^ cells in isograft and allograft groups, respectively (*n* = 6). ***P < 0.001; Values are showed as mean ± SEM. Statistical analysis was determined by two‐tailed Student's *t*‐test.

It has been previously reported that EVs exude through the vascular endothelial gaps and enter the tissue microenvironment, communicating with distant tissues.^[^ ^20^ ^]^ Meanwhile, EVs have also been reported to exude through the vascular endothelial barrier and enter the renal interstitial microenvironment.^[^ ^21^ ^]^ The immunofluorescence showed that renal cells intensify the expression level of VCAM‐1 and ICAM‐1 stimulated by inflammatory factors, serving as anchor point for effectors adhering to (Figure S12, Supporting Information). As shown in **Figure** **3**A and Figure S13A (Supporting Information), after the stimulation of IL‐1*β* (20 ng mL^−1^) and IFN‐*γ* (50 ng mL^−1^), HK‐2 cells significantly increased the expression levels of ICAM‐1 and VCAM‐1. In line, DiD‐labeled Foe‐TEVs adhered to HK‐2 cells were elevated from 10.38% ± 1.14% to 24.98% ± 1.27%, however, the positive area of DiD‐labeled Foe‐TEVs could decrease with the ICAM‐1/VCAM‐1 monoclonal antibody blocking. Moreover, using the STED super resolution confocal microscopy to image the interactions between Foe‐TEVs and the targeting cells, it was shown that EVs engineered with abundant integrin adhere to ligands of VCAM‐1/ICAM‐1 on the plasma membrane, forming the contact dependent immune regulation. At the same time, we observed a large number of EVs insides the cells, implying the uptake of EVs for cargo delivery (Figure S13, Supporting Information). Next, to further confirm the engineered integrins medicated EVs adherence, we have performed the knockout and blocking experiment of integrins in Foe‐TEVs and assay the retention of EVs in grafts and secondary lymphoid organs (Figure S14, Supporting Information). Considering integrins is obligate heterodimers composed of *α* and *β* subunits and there are multiple isoforms of these subunits, Itgb2 knockout (Itgb2^−/−^) mice were utilized to collect Itgb2 knockout Foe‐TEVs.^[^ ^22^ ^]^ Additionally, EPR20825 (Abcam, ab210515) antibody was performed as integrin blocking, which cross‐reacts with more than one of the integrin beta subunits. In vitro, the microfluidic chip was used to simulate the process of EVs adhesion to tubular epithelial cells. DiD‐labeled Foe‐TEVs adhering to HK‐2 cells in Itgb2^−/−^ group and EPR20825 blocking group showed a remarkable decrease compared with Foe‐TEVs group. In vivo, it has been observed that Itgb2 knockout and EPR20825 blocking effectively inhibited the recruitment and adhesion of EVs to inflammatory sites of grafts. Studies have assessed the vital role of secondary lymphoid organs, especially the spleen and lymph node in transplantation, as they are crucial locations for alloantigen recognition and processing.^[^ ^23^ ^]^ In order to demonstrate the immune adherence of Foe‐TEVs, the fluorescence in allograft, spleen, ADLNs, and other organs was measured via the IVIS Spectrum imaging system. It was found that among these samples, the fluorescence was mainly presented in the liver, spleen, allograft, and ADLNs, with only limited amounts in the heart and lung (Figure 3B). Whereas, kidney fluorescence in the Foe‐TEV group was at least more than three times as much as the Vector‐TEVs group, but gradually decreased over time, peaking at 48 h after performing the allotransplantation. In line, the fluorescence in the Foe‐TEV group of two other regions of adaptive immunity, the spleen and ADLN, also peaked 48 h after transplantation, which was more than twice that of the Vector‐TEVs group (Figure 3C). Importantly, the residual radiance of the injection of dye alone was significantly lower compared to the Foe‐TEVs group, with levels of 0.61 ± 0.05, 1.25 ± 0.16, and 0.62 ± 0.06 × 10^9^ p s^−1^ cm^−2^ sr^−1^ in spleen, renal allograft, and ADLNs, respectively, indicating targeting and retention effect of EVs engineering strategy. Furthermore, the bioluminescent labeling of EVs using green fluorescent protein fused to CD63 sequence has been provided to achieve an accurate tracking of EVs. Under the 488 nm excitation light, the bioluminescence of EVs gradually enhanced with the concentration increasing and presented obvious fluorescence at 10^9^ particles. After the injection with Foe‐TEVs, fluorescence was found to distribute in the spleen, kidney allograft, and ADLNs and the accumulation of Foe‐TEVs was increased gradually, peaking at 48 h after injection. At the peaking time, the emGFP positive percent of spleen, allograft, and ADLNs were 65.47% ± 5.69%, 49.56% ± 4.11%, and 27.86% ± 1.69%, respectively, which were more than twice the Vector‐TEVs group. At later time point, the intensity of EVs significantly decreased in the allografts and displayed an obvious clearance effect. However, at 192 h postinjection, the remaining positive area in Foe‐TEVs with level of 4.83% ± 0.37% remained significantly higher than that of the Vector‐TEVs with only 1.51% ± 0.02% (Figure S15, Supporting Information). Given that considerable numbers of Foe‐TEVs accumulated in spleen, allograft and ADLNs compared with PBS and Vector‐TEVs groups, we postulated that Foe‐TEVs decorated with integrin molecules might effectively locate these organs through immune adhesion. Evaluation of effective adhesion via immunofluorescence staining also confirmed that Foe‐TEVs were more densely distributed in the allograft than the isograft group, whereby the DiD^+^ ratio increased from 2.56% ± 0.31% to 23.82% ± 2.11%, and also achieved efficient binding with the endothelium, TECs, and glomerular sites of allogeneic immune injury in the allograft (Figure 3D). Then, a study on the mechanism related to immune adhesion of Foe‐TEVs was conducted. We enucleated and lysed transplanted kidneys from the allograft group and the isograft group, to generate single‐cell suspensions for quantitative monitoring of DiD adhered cells by flow cytometric analysis. For CD54, consisting of five extracellular Ig domains, integrins *α*_L_ *β*_2_ mediated adhesion occurs upon immune stimulation, while CD106 is recognized by integrins *α*_4_ *β*_1_ and mediates adhesion.^[^ ^24^ ^]^ Results revealed that Foe‐TEVs^+^ CD54^+^ cells in the allograft group increased noticeably, and the average proportions of double‐positive cells in these two groups were 23.67% ± 1.66% and 56.47% ± 3.13%, respectively (Figure 3E). Consistently, the average proportions of Foe‐TEVs^+^ CD106^+^ cells in the allograft group were 69.32% ± 5.77%, while 9.18% ± 0.81% in isograft group (Figure 3F).

### 2.4 The Immune Adhesion of Foe‐TEVs via Enhanced Subcapsular Capturing and Effector Interacting

**Figure 4:**
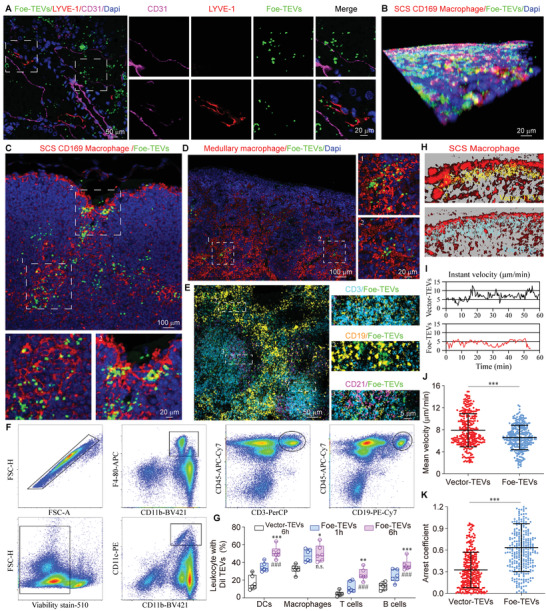
The immune adhesion of Foe‐TEVs via enhanced subcapsular capturing and effector interacting. A) The distribution of Foe‐TEVs in lymphatic vessels and blood vessels presented by immunofluorescence staining in allograft. B) Confocal imaging of Foe‐TEVs distribution in subcapsular sinus of draining lymph node. C) The distribution of Foe‐TEVs under the capsule and in the corticomedullary junction shown by multiple immunofluorescence staining. D) The interaction between Foe‐TEVs and medullary macrophage in the medulla shown by multiple immunofluorescence staining. E) The interaction of Foe‐TEVs with T cells (CD3^+^), marginal zone B cells (CD19^+^), and follicular B cells (CD21^+^) in germinal centers. F) Flow cytometry analysis of adhesion of Foe‐TEVs to CD11b^+^CD11c^+^DCs, F4‐80^+^macrophages, CD45^+^CD3^+^T cells, and CD45^+^CD19^+^B cells. G) Quantification of leukocyte percentage with Dil^+^ TEVs 1 and 6 h after intravenous injection of Vector‐TEVs or Foe‐TEVs (*n* = 6). *P < 0.05, **P < 0.01, and ***P < 0.001 compared to the Vector‐TEVs 6 h group; ^###^ *P* < 0.001 compared to the Foe‐TEVs 1 h group. H) Confocal images showing tracks of Foe‐TEVs and Vector‐TEVs contacting with SCS macrophages under subcapsular sinus of lymph nodes. I) The instant velocity of individual Vector‐TEVs and Foe‐TEVs. J) The mean velocity of Vector‐TEVs and Foe‐TEVs under subcapsular sinus of lymph nodes (*n* = 300). K) The arrest coefficient of Vector‐TEVs and Foe‐TEVs (*n* = 300). ***P < 0.001. Values are presented as mean ± SEM. Statistical analysis was determined by two‐tailed Student's *t*‐test.

The antigen in the graft is recognized and transported by the donor APCs and drained to lymph nodes and spleen through the lymphatic vessels to stimulate systemic rejection.^[^ ^25^ ^]^ In this case, the incoming antigen is first filtered and processed under the subcapsular and further activates T and B cell response.^[^ ^26^ ^]^ To verify whether the Foe‐TEVs can effectively regulate the immune contact with the effector cells and inhibit graft rejection, fluorescently labeled Foe‐TEVs were administered intravenously and were evaluated by immunofluorescence staining. Representative confocal images showed that Foe‐TEVs were distributed in and around CD31‐labeled vessels and LYVE‐1‐labeled lymphatic vessels, and more concentrated around lymphatic vessels 6 h after injection in the renal allograft, suggesting that Foe‐TEVs can enter lymphatic drainage (**Figure** **4**A). In regions that produced adaptive immunity, CD169^+^ macrophages exist in the subcapsular sinus (SCS) of lymph nodes and are the frontline of immune defense for processing and draining lymph fluid and provide key signals for antigen information presentation and germinal center activation.^[^ ^27^ ^]^ CD169 is a member of the Sialic acid‐binding IgG‐like lectin family, expressed on the surface of specific macrophage subsets. SCS CD169^+^ macrophages could collaborate with DCs to initiate effective CD8^+^ T cell responses as well as activate B cells for adaptive immunity.^[^ ^28^ ^]^ Confocal imaging of Foe‐TEVs distribution suggests that Foe‐TEVs rapidly developed interactions with SCS CD169^+^ macrophages after entering the immune activated graft draining lymph nodes, as indicated by the apparent double positive signal of macrophages with fluorescently labeled Foe‐TEVs under the tunica (Figure 4B). Microscopic imaging technology shows that after antigen exposure to ADLNs, SCS CD169^+^ macrophages adhered to and phagocytized some vesicles under the lymph node envelope, and that Foe‐TEVs were distributed under the capsule and in the corticomedullary junction (Figure 4C). Similarly, in the medulla, we also observed the immune adhesion between Foe‐TEVs and medullary macrophages (Figure 4D). Considering that Foe‐TEVs are rich in integrin molecules, we further explored whether they could achieve immune adhesion with other immune cells. As expected, in germinal centers deeper into lymph nodes, Foe‐TEVs can interact with T cells (CD3^+^), marginal zone B cells (CD19^+^), and follicular B cells (CD21^+^) (Figure 4E). Meanwhile, the results of flow cytometry confirmed that Foe‐TEVs could significantly interact with CD11c^+^ DCs, CD11b^+^ F4/80^+^ macrophages, CD3^+^ T cells, and CD19^+^ B cells (Figure 4F). The statistical results showed that the adhesion rate of Foe‐TEVs to the above cells was markedly higher than the Vector‐TEVs group, and increased with the passage of time. After 6 h of treatment, the proportion of DCs, macrophages, T cells, and B cells adhered with Foe‐TEVs were 49.79% ± 3.94%, 47.38% ± 5.62%, 21.73% ± 2.04%, and 34.83% ± 2.17%, respectively (Figure 4G). To further assay the spatial disseminating process of Foe‐TEVs, we determined the instantaneous velocity of single Vector‐TEVs, Foe‐TEVs, Itgb2^−/−^ Foe‐TEVs, and EPR20825 blocking Foe‐TEVs under the subcapsular sinus of lymph nodes by confocal tracking (Figure 4H; Figure S14, Supporting Information). The statistical results showed that the instantaneous velocity peak of Vector‐TEVs was higher than that of Foe‐TEVs (Figure 4I). Additionally, the mean velocity of Vector‐TEVs, Foe‐TEVs, Itgb2^−/−^ Foe‐TEVs, and EPR20825 blocking Foe‐TEVs entering under the lymph node capsule were recorded (Figure 4J,K; Figure S14, Supporting Information). Each point corresponds to an effectively moving vesicle, and the mean velocity of Foe‐TEVs was significantly lower than that of Vector‐TEVs, Itgb2^−/−^ Foe‐TEVs, and EPR20825 blocking Foe‐TEVs, while with a higher arrest coefficient. Moreover, the allosensitization kinetics and kidney injury molecular‐1 staining confirmed that Itgb2^−/−^ group and EPR20825 blocking group possessed lower immunosuppression and graft protection effects than the Foe‐TEVs. The above results further support that Foe‐TEVs realized the effective adhesion and contacting interaction with target cells due to abundant integrins.

### 2.5 Foe‐TEVs Attenuated Excessive Allosensitization Kinetics in Secondary Lymphoid Organ

**Figure 5:**
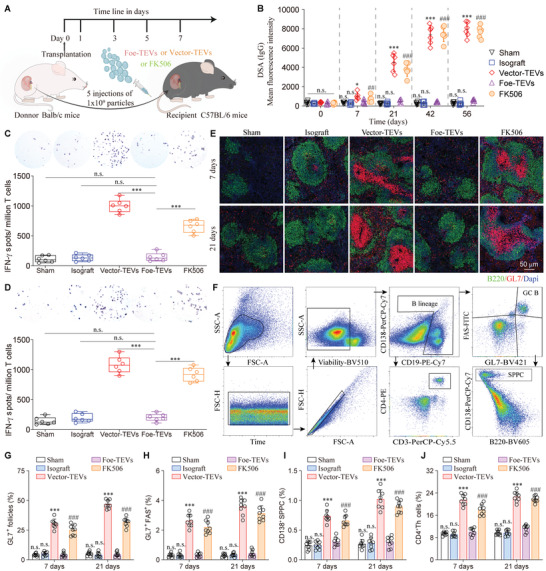
Foe‐TEVs attenuated excessive allosensitization kinetics in secondary lymphoid organ. A) Schematic diagram revealing the administration of EVs in sham, isograft, Vector‐TEVs, Foe‐TEVs, and FK506 groups. B) MFI detection of total DSA levels in serum at day 0, day 7, day 21, day 42, and day 56 post‐transplant, respectively (*n* = 6). C) Kinetics of allospecific T cell responses 7 days post‐transplant (*n* = 6). D) Kinetics of allospecific T cell responses 21 days post‐transplant (*n* = 6). E) immunofluorescence staining of GL7^+^ GCs located within the follicle (B220^+^) in spleen at 7 days and 21 days post‐transplant, respectively. F) Gating strategy for isolating GL7^+^ FAS^+^ GCB cells, CD138^+^ SPPCs, and CD4^+^ Th cells. G) The quantitative statistics of GL7+ germinal centers in E) at day 7 and day 21 post‐transplant (*n* = 8). H–J) The quantitative statistics of GL7^+^ FAS^+^ GCB cells (H), CD138^+^ SPPCs (I), and CD4^+^ Th cells (J) at day 7 and day 21 post‐transplant respectively (*n* = 8). DSA, donor specific antibody; GC, germinal center; SPPC, splenic plasma cell; n.s., not significant, ***P < 0.001, ###*P* < 0.001 compared with the Foe‐TEVs group. Values are presented as mean ± SEM. Statistical analysis was determined by two‐tailed Student's *t*‐test.

We established a renal rejection model with Balb/c (H‐2d) mice as graft donors and C57BL/6 (H‐2b) mice as recipients with simultaneous humoral and cell‐mediated pathological injury. In this case, we generated five groups: sham, isograft, Vector‐TEVs, Foe‐TEVs, and FK506 treated group, which inhibited development and proliferation of T cells commonly used in clinic (**Figure** **5**A). Graft antigens are drained into adaptive immune organs, where antigen recognition occurs. Under the stimulation of antigen, B cells form germinal center, while T cells form adaptive activation response.^[^ ^29^ ^]^ DSA was reported as a promising biomarker predicting graft survival.^[^ ^30^ ^]^ In order to assay the effects compared to Vector‐TEVs and KF506, a control group of the allograft without any treatment was included (Figure S16, Supporting Information). It was noted that the serum level of DSA elevated with the increase of postoperative time in the allograft group. A similar trend could also be found of germinal center formation and GL7^+^ follicle percentage in the spleen. Meanwhile, it was shown that the allograft possessed the histological features of chronic rejection including glomerulonephritis, tubulitis, and interstitial fibrosis, which was not difference compared to Vector‐TEVs group at 8 weeks post‐transplant. In the study, it seemed that the allosensitization kinetics was improved with the treatment FK506, however, it was not superior to the allograft and Vector‐TEVs group at the study endpoint showing no more than 20% survival rate of mice at week 12 after transplantation. It was found that the titer of de novo DSA in serum significantly decreased by 92.38% ± 8.65% after Foe‐TEVs treatment comparing to the Vector‐TEVs group at 56 days and was maintained at a baseline level close to nondetected (Figure 5B). We evaluated the kinetics of T cell alloresponse after transplantation of allograft. After stimulation with the irradiated spleen cells from the donor, a considerable number of IFN‐*γ* ‐secreting T cells were detected in the Vector‐TEVs treated group, suggesting the typical pathological reaction of cellular rejection stimulated by alloantigen. ELISPOT assays showed that both Foe‐TEVs and FK506 inhibited allosensitization of directly alloreactive T cells, whereby FK506 showed only 36.37% inhibitory effect and Foe‐TEVs decreased to the baseline level. Despite the potent inhibition in Foe‐TEVs and FK506, the inhibitory effect of FK506 decreased with the passage of time sharply (Figure 5C,D). Migration of B cells to follicles triggers a germinal center (GC) response accompanied by the development of classical secondary follicles, and antibody secreting plasma cells (CD138^+^) are produced by GL7^+^ activated B cells to promote humoral immune responses. Immunofluorescence analysis revealed 48.58% ± 5.01% of B cell follicles exhibiting a GL7^+^ activated phenotype in Vector‐TEVs group, meanwhile GL7^+^ follicles fell to low levels at 7 and 21 days after Foe‐TEVs treatment (Figure 5E,G). Next, we evaluated antibody mediated rejection by quantification of the GC B cells, spleen plasma cells (SPPCs) and the CD4^+^ Th cells (Figure 5F). Specifically, the total B‐lineage cells containing both CD19^+^ cells and CD138^+^ plasma cells were used for quantifying frequencies of GCs and SPPCs. There were no significant differences in GC formation among sham, isograft and Foe‐TEVs groups. The infiltration of CLT^+^FAS^+^, CD138^+^ SPPC, and CD4^+^ Th cells decreased to a low level after Foe‐TEVs treatment at 21 days post‐transplant in ADLNs (Figure 5H–J). To illustrate the dosage–effect relation, we administered different doses of FK506 including 1 and 5 mg kg^−1^ (Figure S17, Supporting Information). It was noted that FK506‐5 mg had a better performance in suppressing allospecific T cell responses, which decreased by 20% compared with FK506‐1 mg group at 7 and 21 days post‐transplant, however, it was still more than five times higher than Foe‐TEVs group. Meanwhile, the serum level of DSA in FK506‐5 mg group decreased slightly compared with FK506‐1 mg group at day 7, day 21, and day 42 but remained significantly higher than Foe‐TEVs group. A similar trend could also be found of germinal center formation in the spleen. Therefore, it seemed that the therapeutic efficacy was improved with the increase of FK506, whereas it remained significantly inferior to the Foe‐TEVs group at each point of study, further suggesting that adhesive Foe‐TEVs exhibiting comprehensive immunosuppression by contacting and secretory dependent immunosuppressive approaches. For the treatment of solid organ transplantation rejection, the application of EVs is mainly focused on the proliferation and activation of T cells, however, the immunoregulatory strategy of B cells remains a challenge.^[^ ^31^ ^]^ Evidently, the above results verified that through immune contact, Foe‐TEVs lowered allosensitization by suppressing alloreactive T cell response (assessed by ELISPOT) as well as B cells activation (measured by concentration of DSAs in serum and GC and plasma cell formation profile) in the spleen, further suggesting the potential of immune adhesive Foe‐TEVs in graft rejection.

### 2.6 Foe‐TEVs Improved Inflammatory Infiltration and Biased Polarization in Renal Allograft

**Figure 6:**
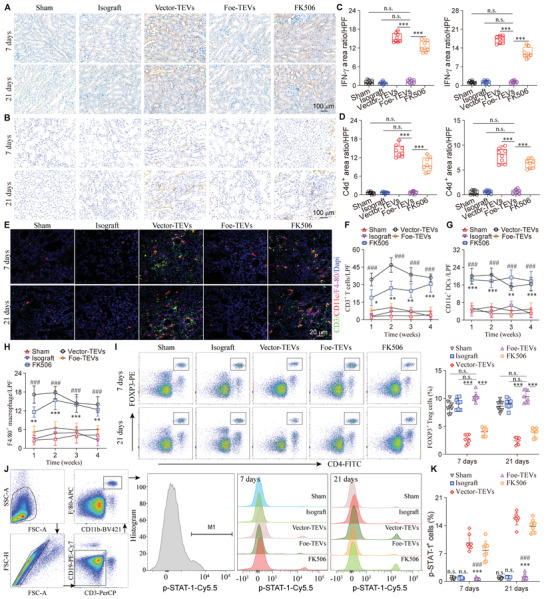
Foe‐TEVs improved inflammatory infiltration and biased polarization in renal allograft. A) Immunohistochemistry of IFN‐*γ* in sham, isograft, Vector‐TEVs, Foe‐TEVs, and FK506 groups at 7 days and 21 days post‐transplant. B) Immunohistochemistry of C4d in sham, isograft, Vector‐TEVs, Foe‐TEVs, and FK506 groups at 7 days and 21 days post‐transplant. C) Quantification of IFN‐*γ* positive ratio in (A) (*n* = 8). D) Quantification of C4d positive ratio in (B) (*n* = 8). E) Representative immunofluorescence staining of F4/80^+^ macrophages, CD3^+^ T cells as well as CD11c^+^ DCs in the kidney at 7 days and 21 days post‐transplant. F–H) Quantification of the infiltration of T cells, DCs, and macrophages in the kidney (*n* = 6). *P < 0.05, **P < 0.01, ***P < 0.001, ^###^ *P* < 0.001, compared with the Foe‐TEVs. I) Representative plots and quantitative analysis of FOXP3^+^ T cells in the kidney of different groups with flow cytometry at 7 days and 21 days post‐transplant (*n* = 8). J) Flow histogram showing the number of p‐STAT‐1^+^ macrophages in graft tissues at 7 days and 21 days post‐transplant. K) Quantitative assay of p‐STAT‐1^+^ polarized macrophages in renal allograft (*n* = 8). n.s., not significant compared to the Foe‐TEVs group; ***P < 0.001 compared to the Vector‐TEVs group; ^###^ *P* < 0.001 compared to the FK506 group. Values are presented as mean ± SEM. Statistical analysis was performed by two‐tailed Student's *t*‐test.

IFN‐*γ* is an endogenously produced Th1 cytokine, which activates and promotes lymphocyte functions as a positive regulator in immune rejection, participating in the promotion of graft rejection.^[^ ^32^ ^]^ Thus, immunohistochemistry was carried out to examine the deposition of IFN‐*γ* in graft tissues. The Foe‐TEVs treatment effectively alleviated IFN‐*γ* intrarenal deposition from 16.94 ± 1.76 to 12.59 ± 1.31 (**Figure** **6**A,C). Additionally, as a paramount degradation product of the classic complement pathway, C4d was recognized as an independent risk factor in allograft failure, associating with the abrupt decline of graft function.^[^ ^33^ ^]^ Immunohistochemistry showed that Foe‐TEVs also significantly reduced the deposition of C4d (Figure 6B,D). However, Foe‐TEVs significantly decreased to the baseline level instead of partially inhibiting the accumulation of C4d as in FK506 group. Then, we evaluated the recruitment of major immune cells in the graft by confocal immunofluorescence (Figure 6E). It was revealed that FK560 possesses an inhibitory effect on the infiltration of CD3^+^ T cells in allografts, but Foe‐TEVs are even more inhibitory (Figure 6F). Moreover, Foe‐TEVs also significantly reduced the infiltration of CD11c^+^ DCs and F4/80^+^ macrophages, which are the unique therapeutic effect of Foe‐TEVs compared to FK560 (Figure 6G,H). During the progression of allograft rejection, the infiltration of FOXP3^+^ Treg cells was significantly diminished aggravating immune response.^[^ ^34^ ^]^ Nevertheless, flow analysis results showed that immune adhesive Foe‐TEVs effectively reversed the above changes of infiltration of CD3^+^ T cells, CD11c^+^ DCs, and F4/80^+^ macrophages. With a view to the negative regulation of graft rejection, CD4^+^FOXP3^+^ Treg cells emerged with a threefold increase after Foe‐TEVs treatment at 7 days and 21 days post‐transplant compared to the Vector‐TEVs group (Figure 6I). Macrophages abounded at the site of the graft and correlated with the occurrence of the graft rejection.^[^ ^35^ ^]^ We also quantified the number and the distribution of proinflammatory M1 polarized macrophages. After treating with immune adhesive Foe‐TEVs, the infiltration of p‐STAT‐1^+^ macrophages decreased from 17.56% ± 2.31% to 2.45% ± 0.31% (Figure 6J,K). Moreover, the percentage of M1 macrophages gradually reduced over time, which was lower in Foe‐TEVs and FK506 groups, particularly the Foe‐TEVs group, on days 14 and 28 compared to the Vector‐TEVs group. To conclude, adhesive Foe‐TEVs with contact‐dependent and secretory effectors alleviate allograft rejection from multiple levels, by inhibiting IFN‐*γ* and C4d deposition, alloreactive immune cells infiltration and type 1 biased immune polarization, while increasing the proportion of Treg cells.

### 2.7 Foe‐TEVs Improved Renal Fibrosis, Graft Function, and the Survival Period of Recipients

**Figure 7:**
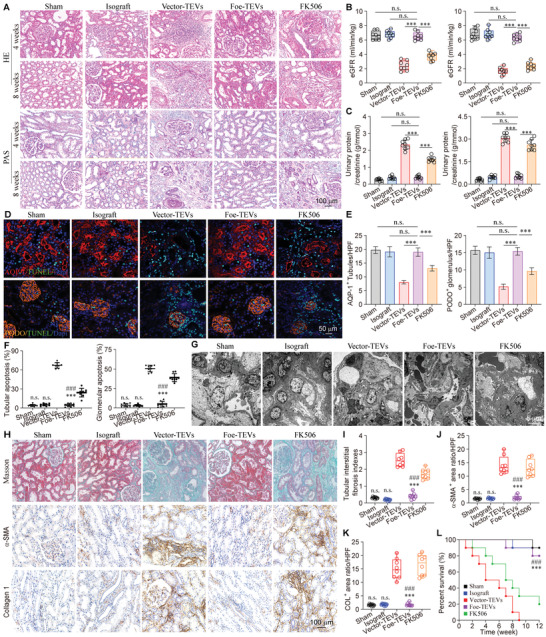
Foe‐TEVs improved the renal fibrosis, graft function, and survival period of recipients. A) Representative images of H&E and PAS staining of glomerulus as well as renal tubules in sham, isograft, Vector‐TEVs, Foe‐TEVs, and FK506 groups after the 4th and the 8th week post operation. B) The estimation of GFR in each group 4th (left) and the 8th week (right) after transplantation (*n* = 8). C) Analysis of urinary protein/creatinine ratio in each group the 4th (left) and the 8th (right) week post operation (*n* = 8). D) Immunofluorescence of AQP‐1, Podocalyxin, and TUNEL showing the morphology and distribution of renal tubules and glomerulus in each group after the 8th week postoperative. E) Quantitative fluorescence intensity of AQP‐1^+^ tubules and PODO^+^ glomerulus (*n* = 8). F) Quantification of the apoptotic cells of glomerulus and renal tubules respectively (*n* = 8). G) TEM images of the ultrastructure of allograft tissue after the 8th week postoperative. H) Masson staining and representative immunohistochemical evaluation of *α*‐SMA and collagen I after 8th week postoperative. I) Fibrotic index of renal interstitial fibrosis 8 weeks postoperation (*n* = 8). J–K) The analysis of *α*‐SMA and type I Collagen positive area ratio in each group after 8th week postoperative (*n* = 8). L) Survival percentage of allograft recipients with different treatments (*n* = 6). n.s., not significant compared to the Foe‐TEVs group; ***P < 0.001 compared to the Vector‐TEVs group; ###*P* < 0.001 compared to the FK506 group. Values are presented as mean ± SEM. Statistical analysis was determined by two‐tailed Student's *t*‐test.

To further elucidate the effects of Foe‐TEVs in transplantation, morphological and functional analyses of renal allografts were conducted. Hematoxylin and eosin (H&E) staining and PAS staining in the Vector‐TEVs group revealed considerable lymphocyte infiltration at 4 weeks after operation. At 8 weeks, the vascular endothelial cells proliferated and the lumen was narrow, which is a typical pathological injury of rejection.^[^ ^36^ ^]^ After treatment with Foe‐TEVs, most glomerulus and renal tubules maintained a normal structure, similar to the sham group and the isograft group (**Figure** **7**A). It was noted that glomerular filtration rate (GFR) was elevated more than twofold with Foe‐TEVs treatment both at week 4 and 8 post‐transplant compared with Vector‐TEVs treatment. In line, compared with the Vector‐TEVs treatment, urinary protein/creatinine ratio revealed a remarkable decrease in the Foe‐TEVs treated recipient mice (Figure 7B,C). Glomerulus and renal tubules were then labeled by immunofluorescence and it was found that Foe‐TEVs treatment appeared to improve the distributional density of renal parenchyma. Quantitative analysis also demonstrated that the numbers of glomerulus and renal tubules were well preserved in Foe‐TEVs group. Meanwhile, the apoptotic cells were significantly reduced after Foe‐TEVs treatment compared with Vector‐TEVs group and FK506 group (Figure 7D–F). Electron microscopy showed that Foe‐TEVs alleviated typical pathological changes such as foot process fusion as well as glomerular basement membrane thickening in renal rejection (Figure 7G). Through immunohistochemical staining, alleviated interstitial fibrosis was identified in the Foe‐TEVs treated group. It was demonstrated that the interstitial fibrosis index of grafts (0.43 ± 0.02) in the Foe‐TEVs treated group at 8 weeks post‐transplant was decreased compared with Vector‐TEVs (2.39 ± 0.18), but no significant difference was identified compared to the sham group and isograft group. Following Foe‐TEVs treatment, the tubular atrophy and glomerulosclerosis were significantly alleviated, as the type I Collagen positive area ratio and *α*‐SMA positive area ratio were decreased dramatically (Figure 7H–K). These quantitative changes revealed that immune adhesive Foe‐TEVs effectively protected the renal functions from impairments, providing a beneficial basis for graft survival.^[^ ^37^ ^]^ Consistently, it was shown that delivery of Foe‐TEVs significantly improved the survival rate of mice (83.56%) compared to the Vector‐TEVs (0%) and FK506 (20.07%) groups at 12 weeks (Figure 7L).

## 3 Conclusion

In summary, we genetically engineered extracellular vesicles with the selected transcript of FOXP3, inspired by natural Treg cells. With limited exogenous gene stress, the Foe‐TEVs simultaneously displayed with adhesive molecules, secretory cytokines as well as coinhibitory contact molecules involving CTLA‐4 and PD‐1. We showed that Foe‐TEVs effectively targeted to the regions of allogeneic immune injury and reversed the tracking velocity of vesicles to a retained phenotype and enhanced arrest coefficient under drainage fluid in the secondary lymphoid tissue. The alloreactive responses after transplantation were alleviated efficiently, by suppressing the biased polarization of Th1 cells, depressing DSA production, and hindering complement activation. Furthermore, immune adhesive Foe‐TEVs attenuated pathological responses, restored renal function, and prolonged the survival period of the recipient in mice. Our findings provide a novel insight and potential for the clinical translation of adhesive engineered extracellular vesicles edited by selected transcript, providing a promising synergetic strategy for the treatment of graft rejection.

## 4 Experimental Section

### Isolation and Culture of Th Cells

The spleen was taken from the scarified mice and cut into small pieces of 5–10 mm^3^ for grinding on a 200‐mesh metal filter net, rinsed repeatedly with RPMI 1640. The filtered liquid was added to the centrifuge tube containing the mouse lymphocyte solution and centrifuged at 1500 r min^−1^ for 15 min. The creamy white mononuclear cell layer was taken to obtain mononuclear cells, which were added to MACS buffer and centrifuged at 1000 r min^−1^ for 10 min twice, following being suspended in MACS buffer. Afterward, splenic Th cells were isolated by applicating a magnetic‐based Treg Isolation kit (Miltenyi Biotech). The purity of Th cells reached 95.21% after isolated by immunomagnetic beads, as shown in Figure S1 of the Supporting Information. Next, purified Th cells were cultured with advanced RPMI 1640 medium in 24‐well plates and stimulated with plate‐bound‐anti‐CD3 (5 µg mL^−1^) as well as soluble anti‐CD28 (5 µg mL^−1^) accompanied with recombinant mouse IL‐2 (100 U mL^−1^) for 4 days.

### Preparation of Foe‐Th

For the preparation of Foe‐Th, the first step was to construct lentivirus vectors. FOXP3 gene was subcloned into lentiviral vectors and then the Foe‐Th cells of inducible lentivector containing reporter element GFP was constructed. Next, Foe‐Th cells inducible lentivectors were transfected into 293T cells (4 × 10^6^), together with envelope plasmids as well as packaging plasmids. After transfection for 24 and 48 h, fluorescence microscopy was used to detect the expression of GFP and observe the transfer efficiency. Then, the supernatants were harvested with a 0.45 µm filter, and then the viral particles were collected by centrifuging at 25 000 rpm for 2 h at 48 and 72 h after transfection. Using RPMI 1640 medium, the viral pellets were resuspended and the viral titer was estimated through Broth dilution method. After the preparation of lentivirus vectors, prepared Th cells were transfected by lentivirus for Foe‐Th cells obtainment. Th cells (1.5 × 10^6^) were seeded onto a six‐well culture plate accompanied with 50 U mL^−1^ recombinant mouse IL‐2, and FOXP3 gene package concentrated in the lentivirus venom was added to the plate. The medium was replaced after 72 h, and then cells were cultured in an incubator with 50 U mL^−1^ recombinant mouse IL‐2 at 37 °C for 96 h. Th cells transfected with FOXP3 gene packaged lentivirus were the Foe‐Th cells, and were further isolated through flow cytometry.

### Preparation of EVs

Foe‐Th cells (1 × 10^7^) contained in cultured supernatant and were centrifuged at 300 × *g* (4 °C) to remove cells. Then, cell fragments were discarded via 2000 × *g* centrifugation for 10 min. Furthermore, the debris in the supernatant were discarded at the condition of 10 000 × *g* centrifugation for 30 min. Next, the last supernatant was then ultracentrifuged at 100 000 × *g* for 70 min twice to obtain a pellet containing Foe‐TEVs. The pellets were rinsed with 10 mL normal saline and subjected to ultracentrifugation overnight at 100 000 × *g*. Finally, The Foe‐TEVs (1 × 10^9^ particles) were dispersed in 1 mL PBS keeping at 4 °C for one week. For long‐term Foe‐TEVs storage, they were stored at −80 °C before getting to the next steps.

### TEM

Foe‐TEVs were fixed in 1% PFA, followed by negatively staining with 2% phosphotungstic acid. Afterward, Foe‐TEVs were placed onto a copper grid and processed for drying. Images were captured using a JEM‐1400 TEM (Jeol) an acceleration voltage of 100 kV. Renal tissues were fixed with 4% PFA and were washed gently with distilled water for several times. Then the slices were fixed with 1% OsO_4_ and embedded in OCT. Ultrathin slices were carried out by the LEICA EM UC7 FC7, around 80 nm thickness. Afterward, these sections were stained with lead citrate and uranium acetate. TEM was carried out through the Tecnai G2 SpiritBiotwin at 75 KV.

### NTA

Size and concentration distributions of Foe‐TEVs and Vector‐TEVs were determined via NanoSight NS300 (Malvern, Worcestershire, UK). The camera level was set to 13 (range: 1–16) and the residual settings were arranged automatic mode. Samples were administered and set down under controlled flow, utilizing the Malvern NanoSight syringe pump and script control system. 5 × 30s videos per sample were recorded. The detection threshold was adjusted to 5 and other settings were arranged automatic mode. Nanosight NTA 3.0 software (Malvern, UK) was utilized to analysis the measurements. Samples were run in triplicate, from which distribution, size, and mean concentration of Foe‐TEVs and Vector‐TEVs were computed.

### The Quantification of Protein in Lysed and Unlysed Foe‐TEVs

Foe‐TEVs were added to 50 µL SDS‐Tris buffer heating for 5 min at 95 °C. Then, Foe‐TEVs were placed in a sonication bath for 30 s with 30 s intervals at 4 °C. As for both lysed Foe‐TEVs and unlysed Foe‐TEVs, protein concentrations were determined using BCA according to the manufacturer's instruction (Thermo Fisher Scientific). Moreover, protein lysates of Foe‐TEVs were analyzed utilizing the Quantikine ELISA kit (R&D Systems). Using a PerkinElmer Victor X3 Multilabel Plate Reader, the result was recorded at 450 nm.

### Proteomic Characterization of Cytokines in Foe‐TEVs

Mouse Cytokine Panel A Proteome Profiler antibody arrays (RayBiotech, Norcross, GA, USA) were utilized on the basis of instructions to evaluate the levels of cytokines in the Vector‐TEVs and Foe‐TEVs. Briefly, the proteomic membranes were fixed and developed in respective solutions. Positive signals were captured on developed film utilizing the ChemiDoc Touch Imaging System (Bio‐Rad) and the estimated intensity of each dot was quantified by ImageJ software. These cytokines were screened in the below integrated conditions: Vector‐TEV group comparing with Foe‐TEV group (*p*‐value less than 0.05).

### Natural Treg Cells Preparation and Treg‐EVs Isolation

The spleen single‐cell suspensions were sequentially stained with anti‐CD25 PE (BD, Biosciences) and anti‐PE (BD, Biosciences) magnetic beads on the AutoMACS system (Miltenyi Biotec) for sorting. After that, cells were collected and stained with anti‐CD4 allophycocyanin sorting on an FACS Vantage SE (BD, Biosciences) for the CD25^+^ population. Next, under the stimulation of TGF‐*β* (2 µg mL^−1^) and IL‐2 (100 U mL^−1^), the purified CD4^+^ FOXP3^+^ cells were seeded in EV‐free culture medium at a concentration of 2 million cells per well with 1:5 mouse T‐Activator CD3/CD28 Dyna beads for 2 days. Finally, the supernatant was used for collecting the Treg‐EVs isolation.

### RNA‐Sequencing

An Illumina high‐throughput RNA sequencing experiment was performed to sequence transcriptomic RNA. Briefly, total RNA was collected from the naive Treg cell and Foe‐Th cells. The contamination and degradation of RNA were checking on 1% agarose gels. Agilent 2100 Bioanalyzer (Applied Biosystems) was used to assess the RNA integrity and concentration. Then, the construction of RNA gene library was completed and then was assessed by the Agilent Bioanalyzer 2100 system. Next, gene sequencing was performed using a Novaseq System. Finally, utilizing the DEGSeq R package, differential expression analysis with P 0.05 and log 2 (fold change) of 1 as the threshold for statistical difference expression, was completed.

### Proteomic Analysis

Treg‐EVs and Foe‐TEVs were performed at the dose of 200 µg total proteins using Mass spectrometry analysis. Protein samples were collected by electrophoresis with polyacrylamide gradient gel in SDS running buffer. After rinsing with acetone, the samples were lyophilized and denatured with urea, alkylated with 100 mm iodoacetamide and reduced with 10 mm DTT. Then, using trypsin, it was digested for 16 h at 37 °C. By a reversed phase LC‐MS/MS (VION IMS QTOF, Waters), each sample fractions were analyzed with the content of 10 µg. Briefly, the peptide mixture was loaded onto a column (Acclaim PepMap100 C18, Thermo Fisher Scientific) and separated through the C18 analytical column with mobile phases A (0.1% formic acid) and B (84% acetonitrile and 0.1% formic acid). Utilizing Proteome Discoverer 2.1 (Thermo Fisher Scientific), the raw data were processed to complete identification and comparison the results setting the statistical threshold value < 0.01.

### Western Blot

Protein lysates of Vector‐TEVs, Foe‐TEVs or Foe‐Th cells were prepared according to standard procedures, and the contents of proteins were assessed by the BCA protein concentration measurement kit (Thermo Fisher Scientific). Subsequently, the samples were isolated using Bis‐Tris gel (Invitrogen), and then transferred to polyvinylidene difluoride membranes (Millipore). The membrane was blocked in TBS‐T in 5% BSA for 1 h, incubated with primary antibodies overnight at 4 °C, then rinsing the membranes with PBS. Then, the membranes were covered with secondary antibody. Incubated at room temperature for 2 h, signals were assessed by enhanced Chemiluminescence Advanced System (GE Healthcare). The primary antibodies involving against CD9 (1:1000, Abcam), CD63 (1:1000, Abcam), Tubulin (1:1000, Abcam), TSG101(1:1000, Abcam), CTLA‐4 (1:1000, Abcam), TGF‐*β* (1:1000, Abcam), IL‐10 (1:1000, Abcam), PDCD‐1 (1:1000, Abcam), CD35 (1:1000, Abcam), CD73 (1:1000, Abcam), LAG3 (1:1000, Abcam), TIGIT (1:1000, Abcam), calnexin (1:1000, Abcam), histone 3 (1:1000, Abcam), and GM13 (1:1000, Abcam), ITGA4 (1:800, Abcam), ITGAL (1:800, Abcam), ITGB1 (1:800, Abcam), ITGB2 (1:800, Abcam) and secondary antibody Alexa Fluor Plus 800 (1:10 000, Thermos) were used.

### ELISA

The contents of cytokines including IL‐10, TGF‐*β*, IL‐35, CD73, TGF‐*β*, LAG3, CTLA‐4, PDCD‐1, and TIGIT in Vector‐TEVs and Foe‐TEVs and the concentration of IFN‐*γ* in the cell culture liquid supernatant were determined by the mouse Quantikine ELISA Kit (R&D, Systems). The above experiments were carried out according to the producer's instructions.

### qRT‐PCR

Applying the RNeasy kit (Qiagen), total RNA was separated from cells based on manufacturer's protocols. The purity and quality examination of RNA were detected using spectrophotometry. First strand cDNA was synthesized from extracted RNA with GoScript Reverse Transcription Kit (Promega) and processed to quantitative RT‐PCR (qRT‐PCR) by TaqMan Gene Expression Master Mix (Invitrogen). Subsequently, the levels of miRNAs relative expression were normalized to U6 snRNA and determined with ΔΔCt method. Related primers in this study were detailed in Table S1 of the Supporting Information.

### Northern Blot

Total RNA was isolated from Th cells or Foe‐Th cells with Trizol reagent (Invitrogen). Then the extracted RNA was divided into 30 µg aliquots on a 15% denaturing polyacrylamide gel and then transferred to Genescreen Plus membranes (Perkin‐Elmer). Oligonucleotides complementary to the mature FOXP3 and glyceraldehyde‐3′‐phosphate dehydrogenase (GAPDH) were synthesized and labeled with biotin. For the hybridization, the membranes were incubated overnight with 2 ng mL^−1^ of biotin‐labeled FOXP3 and GAPDH probes. Prehybridization and hybridization were performed utilizing hybridization buffer. The most stringent rinse was performed in 2× SSC and 1% SDS at 37 °C.

### Live/Dead Staining

Suspensions of HUVECs and KFBs were prepared using an assay buffer, with the density at 1 × 10^5^ to 1 × 10^6^ cells mL^−1^. Then, the stained working solution (100 µL) was mixed with cell suspension (200 µL), incubated at room temperature for 15 min. Using a fluorescence microscope, live cells (green) and dead cells (red) were detected concurrently with an excitation filter of 490 ± 10 nm.

### CCK‐8

The CCK‐8 kit (Yeason) was used for cell proliferation assessment. In each well, 500 µL of 2 × 10^5^ count HUVECs were alone or added with Foe‐TEVs or Vector‐TEVs. Similarly, the KFBs were alone or cocultured with different TEVs. It was carefully discarded of the medium from the plate and washed the wells by DMEM three times during the given incubation time. After that, CCK‐8 reagent (40 µL) and DMEM (360 µL) were mixed into the wells and incubated in a 5% CO_2_ incubator for 1 h. A 200 µL of suspension was carefully extracted from each well and placed into a new 96‐well plate. Using the spectrophotometer, absorbance of the cell suspension at 450 nm was measured to evaluate the optical density of living cells.

### Kidney‐Graft Mice Models

To perform renal allograft transplantation, C57BL/6 (H‐2b) mice were utilized as recipients at the age of 8–10 weeks, and the Balb/c (H‐2d) mice were served as donors. The surgical manipulation was carried out as detailed previously.^[^ ^38^ ^]^ In brief, donor and recipient male mice with the weight between 20 and 25 g were anesthetized with 2% isoflurane. The intact left kidney of donor was obtained via isolating ureter from other attachments. Donor kidney was placed in preservation solution at 4 °C for 1.5 h. After removing the left kidney from recipient, renal arteries, veins, and ureters from donor graft and recipient were reconnected using end‐to‐end anastomosis. Recipients were randomly separated into five groups after transplantation involving sham group, isograft group, allograft with the Vector‐TEVs treated group, allograft with Foe‐TEVs treated group, and allograft with FK506 treated group. Recipients were treated with Vector‐TEVs and Foe‐TEVs respectively through intravenous injection for 5 injections of 1 × 10^9^ particles in PBS at day 0, 1, 3, 5, and 7 post‐transplantation. For FK506 group, 1 mg kg^−1^ of FK506 during the first 3 days and every other day until one week. All animals’ procedures were carried out with the review and approve of Animal Protection and Research Advisory Committee of the First Affiliated Hospital, Zhejiang University (4380‐0571‐32).

### Immunocytofluorescence

HK‐2 cells were directly seeded on glass coverslips at low density and then stimulated with IL‐1*β* (20 ng mL^−1^) as well as IFN‐*γ* (50 ng mL^−1^) for 48 h. Subsequently, cells were coincubated with DiD‐labeled Foe‐TEVs for more than 12 h. HK‐2 cells were then fixed for 20 min in 4% paraformaldehyde in PBS as well as permeabilized for 30 min in 0.25% Triton‐X‐100 in PBS containing 5% bovine serum albumin. After that, at 4 °C overnight, primary antibodies against ICAM‐1 (ab282575; 1:500, Abcam) or VCAM‐1 (1:250, Abcam) and samples incubated together and then at 37 °C for 1 h with fluorescence‐labeled secondary antibodies, including Alexa Flour 488 (1:10 000, Abcam) or Cyanine 5 conjugated secondary antibodies (1:250, Abcam) and the nuclei were counterstained with DAPI (Abcam). Images were captured by confocal microscopy (Zeiss LSM 880) and analyzed by ImageJ software (v1.8.0).

### Renal Histology and Immunostainings

Kidneys, hearts, lungs, livers, and spleens were fixed in 4% paraformaldehyde for one day and dehydrated. After paraffin embedding, the slices were collected. The tissue slices were deparaffinized, pretreated, stained with H&E for 10 min, dehydrated in ethanol gradient, air dried, and sealed with resin. The second group of renal slices was also dewaxed. They were stained with Masson's stain in accordance with the supplier's guidelines. Similarly, PAS staining for renal tissue was performed with procedures according to detailed instruction manuals. Finally, all sections were captured with a microscope (Leica, DM1000). As for immunohistochemistry, deparaffinizing as well as rehydrating of the paraffin sections was carried out first, and then antigens were repaired. After set in 3% hydrogen peroxide, sections were incubated at room temperature for 25 min then using serum sealing. Subsequently, sections were incubated with primary antibodies, involving anti‐C4d (1:300, Abcam), anti‐IFN‐*γ* (1:300, Abcam), anti‐*α*‐SMA (1:300, Abcam), and anticollagen I (1:300, Abcam). After washing by PBS, the tissues were covered with secondary antibody (HRP labeled), conjugated with horseradish peroxide. Nuclei were stained with hematoxylin, and staining tissues were observed using a microscope (Leica, DM1000). For Immunofluorescence, kidneys, spleens, and lymph nodes were removed and then fixed in 4% paraformaldehyde for 24 h at 4 °C. Then, above samples were subjected to gradient dehydration, embedded in paraffin and then sliced to 4 µm. Next, the sections were incubated with primary antibodies overnight at 4 °C. Antibodies against CD169 (1:100, Abcam), LYVE‐1 (1:200, Abcam), CD31 (1:200, Abcam), ICAM‐1 (1:100, Abcam), VCAM‐1 (1:100, Abcam), CD3 (1:100, Invitrogen), CD19 (1:100, Abcam), CD21 (1:200, Abcam), B220 (1:200, Abcam), GL7 (1:100, Abcam), CD11c (1:200, Abcam), F4‐80 (1:100, Abcam), AQP1 (1:200, R&D Systems), and Podocalyxin (1:200, R&D Systems). The sections were then incubated with fluorescence‐labeled secondary antibodies, including Alexa 488, Cyanine 3 or Cyanine 5 conjugated secondary antibodies (Abcam), and nuclei were stained with DAPI. Based on the confocal microscope, immunostaining samples were visualized (Carl Zeiss, LSM 880 with Airy Scan).

### In Vivo Fluorescence Imaging

To evaluate in vivo tissue distribution of Foe‐TEVs, PBS, DiD, DiD‐labeled Vector TEVs or DiD‐labeled Foe‐TEVs were injected intravenously into mice after allotransplantation. Mice were euthanized, at 6, 12, 24, 48, 96, and 192 h post injection. Tissues involving the hearts, lungs, livers, spleens, renal allografts, and ADLNs were extracted and imaged by the IVIS Spectrum imaging system (IVIS, Lumina LT Series III).

### Analysis by Two‐Photon Microscopy

Two‐photon microscopy was carried out intravital on ADLNs. Using a nebulized isoflurane and oxygen gas mixture, the mice were anesthetized, which subsequently was fixed on the customized platform. To maintain mice at 37 °C during the procession of intravital imaging, the body temperature of them was monitored using a temperature probe. ADLNs were exposed surgically, embedding them in 2% low‐melting agarose as well as covering them with the coverslip. To visualize the capture of Foe‐TEVs or vector‐TEVs in vivo, DID‐labeled TEVs (1 × 10^9^ particles per injection) were intravenously injected into recipient mice after surgery. To label the SCS macrophages in ADLNs in vivo, 0.25 ug of AF647‐conjugated Ab against CD169 (Biolegend) was injected intravenously for 2 h before imaging. Images were obtained through the Olympus FluoView FV1000 microscope (Olympus America, Center Valley, PA).

### ELISPOT Assays

In accordance with manufacturer's protocol, 96 well ELISPOT plates (MSIPS4510, Millipore) were coated with anticytokine capture monoclonal antibody sterile PBS overnight. These plates were rinsed twice by sterile PBS, closed with PBS containing 1% BSA for 1.5 h, and subsequently rinsed three times by sterile PBS. The purified T cells from Sham or recipient C57BL/6 mice were cultured alone (sham group) or with C57BL/6 DCs (Isograft group) or Balb/c DCs (Allograft groups) (150 000 T cells and 15 000 DCs per well) in the plate precoated with IFN‐*γ* capture antibody at 37 °C for 36 h, respectively. The development plate was incubated with biotin–IFN‐*γ* detection antibody (2 h, room temperature) and then with horseradish peroxidase streptavidin (1 h, room temperature). The computer‐assisted ELISPOT ImmunoSpot image analyzer (Cellular Technology Limited) was utilized to calculate and analyze the spots.

### Allograft Lysate Preparation

The allograft kidney was isolated and lysed with radioimmune precipitation assay buffer (Beyotime, Shanghai, China) containing 1% NP40, 0.1% SDS, 100 µg mL^−1^ PMSF, 1% protease inhibitor cocktail, and 1% phosphatase I and II inhibitor cocktail for 10 min, followed by filtered through a 45 µm strainer in the storage buffer (2% FBS in PBS). Then, the graft grinding supernatants were gathered after centrifugation at 13 000 × *g* for 20 min at 4 °C.

### Detection of Antidonor Ab Concentration

Serum DSA concentrations of C57BL/6 mice were expressed as mean fluorescence intensities of FITC measured by flow cytometry. Briefly, serum was diluted (1:25) and incubated with 1 × 10^6^ C57BL/6 or Balb/c derived splenocytes for 1 h incubation at 4 °C. Then, labeled with FITC‐conjugated goat anti‐mouse IgG antibody, thymocytes were rinsed by cold PBS and fixed in 4% paraformaldehyde‐PBS. Mean fluorescence intensities (IgG) were assessed via flow cytometry.

### TUNEL Staining

To assess renal apoptosis, TUNEL staining was carried out by an In Situ Cell Death Detection Kit‐Fluorescein (Hoffman‐La Roche), as described in the manufacturer's protocol. Briefly, the slices were also stained with AQP‐1 antibody (1:200, R&D Systems) or Podocalyxin antibody (1:200, R&D Systems) for tubular epithelial cells or glomerular cells as well as costained with DAPI (1:10 000).

### Flow Cytometry

Mice were killed and the kidneys, spleens, and renal lymph nodes were collected for flow cytometry assay post‐transplantation. Then the tissues were minced and kidneys were processed with collagenase I (1 mg mL^−1^, Sigma) for single‐cell suspensions, and the spleens and lymph nodes were triturated into single‐cell suspensions. Approximately 1 mL of single‐cell suspensions was taken from each sample, fully added with PBS (containing 0.1% BSA), and then centrifuged for 5 min at 300 × *g*. After lysis red blood cells, anti‐CD 16 and anti‐CD32 antibodies (Abcam) were utilized to block nonspecific binding by incubating cells for 10 min. Subsequently, with a final concentration of 1 × 10^6^ cells, the samples were acquired and the cells were incubated using FACS buffer (PBS with 2% FBS; Invitrogen) for 30 min at 4 °C in darkness. The FACS antibodies used in this article were shown as follows: FITC conjugated to anti‐mouse CD106 (Biolegend, 105705), FITC conjugated to anti‐mouse CD54 (Biolegend, 116105), APC‐Cy7 conjugated to anti‐mouse CD45 (Biolegend, 147707), PerCP conjugated to anti‐mouse CD3 (Biolegend, 100217), APC conjugated to anti‐mouse F4/80 (Biolegend, 123115), PE‐Cy7 conjugated to anti‐mouse CD19 (Biolegend, 152407), BV421 conjugated to anti‐mouse CD11b (Biolegend, 101235), PE conjugated to anti‐mouse CD11c (Biolegend, 117307), FITC conjugated to anti‐mouse FAS (Biolegend, 152605), Bv510 conjugated to FSV (Biolegend, 423117), PerCP‐Cy7 conjugated to anti‐mouse CD138 (Biolegend, 142509), APC conjugated to anti‐mouse CD19 (Biolegend, 152409), PerCP‐Cy5.5 conjugated to anti‐mouse CD3 (Biolegend, 100217), PE‐Cy7 conjugated to anti‐mouse CD4 (Biolegend, 100407), BV421 conjugated to anti‐mouse GL7 (Biolegend, 144613), BV605 conjugated to anti‐mouse B220 (Biolegend, 103243), BV605 conjugated to anti‐mouse CD3 (Biolegend, 100237), PE conjugated to anti‐mouse FOXP3 (Biolegend, 126403), FITC conjugated to anti‐mouse CD4 (Biolegend, 100405), and PerCP‐Cy5.5 conjugated to anti‐mouse p‐STAT‐1 (Biolegend, 666408). Different kinds of cells were captured by Aurora Flow Cytometry (Cytek), and the data analysis was implemented by FlowJo software.

### Biochemical Analysis

Albumin (ALB), Aspartate transaminase (AST), alanine aminotransferase (ALT), and blood urea nitrogen (BUN) were evaluated by an automatic biochemical analyzer (Roche, Germany).

### Measurement of GFR and Albumin/Creatinine Ratio (ACR)

For the assessing of GFR in conscious mice, transdermal GFR technology (MediBeacon Inc., Mannheim, Germany) was applied at prespecified time points. At the time of sacrificing, urine was gathered through bladder puncture, and also the ACR was evaluated as discussed previously.^[^ ^39^ ^]^

### Complete Blood Count Analysis

Whole blood of mouse was collected for Complete Blood Count using the automated blood cell counter, (CD 3700; Abbott Diagnostics, Santa Clara, CA, USA).

### Statistics Analysis

All experiments were replicated for three times and obtained data were presented as mean ± SEM. Data analyzing was performed by the GraphPad Prism (v8.3.0). All statistical tests were comprehensively and clearly illustrated in the parallel figure legends. Using two‐tailed unpaired *t*‐test, the differences between every two groups were analyzed. The one‐way analysis of variance was utilized for the comparison between groups more than two. Partial correlations were utilized to analyze the therapeutical efficacy of Foe‐TEVs treatment in the allograft model. For above comparisons, *P* < 0.05 was presented as statistical significance.

## Conflict of Interest

The authors declare no conflict of interest.

## Supporting information

Supporting Information

## Data Availability

The data that support the findings of this study are available from the corresponding author upon reasonable request.
