# Supplementary material for: Transcript Engineered Extracellular Vesicles Alleviate Alloreactive Dynamics in Renal Transplantation
Source: Adv Sci (Weinh). 2022 Sep 8;9(31):2202633. doi: 10.1002/advs.202202633 (PMC9631077; doi:10.1002/advs.202202633)
Supplement: Supplementary file 1 — Supporting Information [file ADVS-9-2202633-s001.pdf]

ADVANCED SCIENCE
Open Access

Supporting Information

for *Adv. Sci.*, DOI 10.1002/advs.202202633

Transcript Engineered Extracellular Vesicles Alleviate Alloreactive Dynamics in Renal Transplantation

*Jinwen Lin, Junhao Lv, Shiping Yu, Ying Chen, Huiping Wang and Jianghua Chen**

Transcript engineered extracellular vesicles alleviate alloreactive dynamics in renal transplantation

Jinwen Lin, Junhao Lv, Shiping Yu, Ying Chen, Huiping Wang and Jianghua Chen*

Dr. J. Lin, Dr. J. Lv, Dr. S. Yu, Dr. Y. Chen, Dr. H.Wang, Prof. J. Chen

Kidney Disease Center, The First Affiliated Hospital, Zhejiang University School of Medicine. Key Laboratory of Kidney Disease Prevention and Control Technology. National Key Clinical Department of Kidney Diseases. Institute of Nephrology, Zhejiang University. Zhejiang Clinical Research Center of Kidney and Urinary System Disease. Hangzhou, Zhejiang Province, 310003, P. R. China.

Dr. J. Lin,

Zhejiang University-University of Edinburgh Institute, School of Medicine, Zhejiang University. Hangzhou, Zhejiang Province, 310003, P. R. China.

* Corresponding author: E-mail address: chenjianghua@zju.edu.cn (J. Chen).

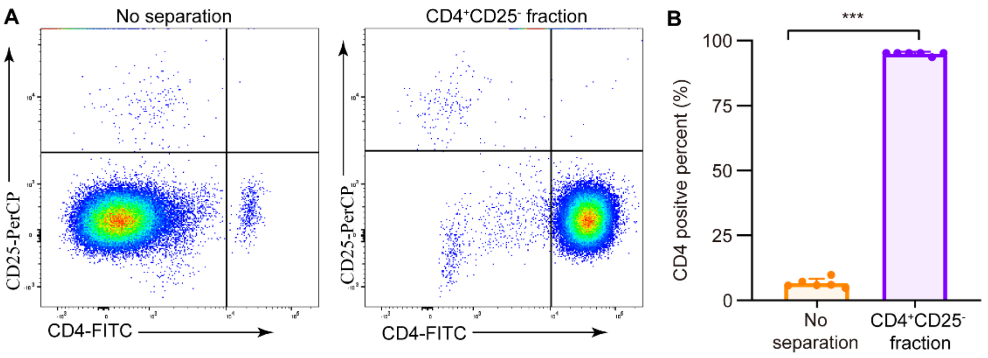

Figure S1. Cell fraction from spleen cells before and after separation. (A) The percentages of $CD4^+$ and $CD25^+$ T cells before and after separation by flow cytometry. (B) The percentages of $CD4^+$ cells (n=6). ***P < 0.001; Values are presented as mean ± SEM. Statistical analysis was performed by two-tailed Student's t test.

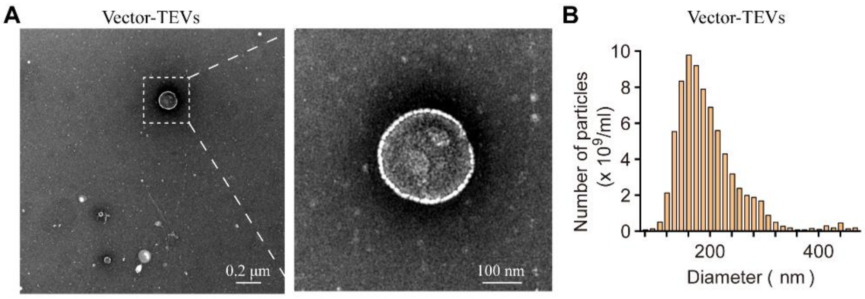

Figure S2. The characterization of Vector-TEVs. (A) Transmission electron microscopy imaging of Vector-TEVs. (B) Nanoparticle tracking analysis of Vector-TEVs.

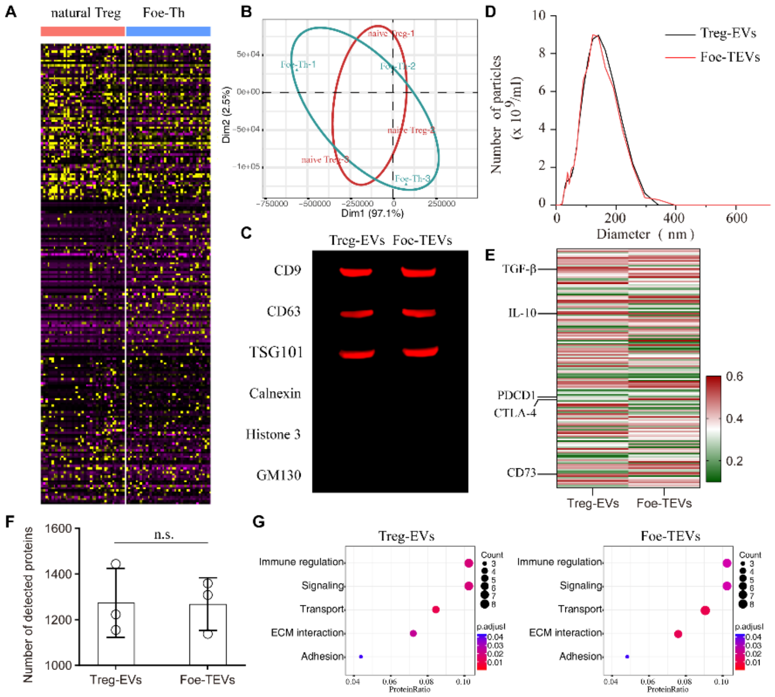

Figure S3. The comparison of natural Treg cell-derived EVs and Foe-TEVs. (A) Heatmap of genes expression between natural Treg cells and Foe-Th cells. (B) Principal component analysis of natural Treg cells and Foe-Th cells, showing conservation of gene expression upon genetic engineering. (C) Western blotting analysis of CD9, CD63 and TSG101, and negative markers of Calnexin, Histone 3 and GM130 expression in natural Treg-EVs and Foe-TEVs. (D) Diameter distribution of Treg-EVs and Foe-TEVs assayed by nanoparticle tracking analysis. (E) A heatmap showing the protein levels of each cytokine obtained from Treg-EVs and Foe-TEVs. (F) The number of total detected proteins in proteomic profiling of Treg-EVs and Foe-TEVs (n=3). (G) Functional enrichment of the proteins identified in Treg-EVs and Foe-TEVs. n.s., not significant; Statistical analysis was determined by two-tailed Student's t test.

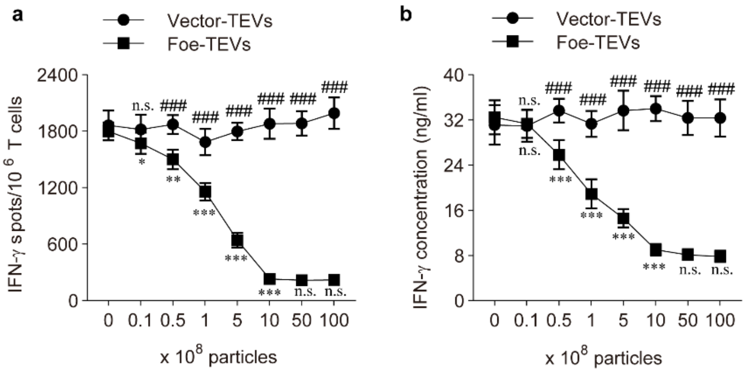

Figure S4. Dose–response curves of effective T cells with varying particles of Vector-TEVs and Foe-TEVs for direct stimulation of DCs derived from allograft doner. (A) IFN-γ spots changes measured by IFN-γ ELISPOT assay. (B) IFN-γ concentration changes measured by ELISA. n.s., not significant; $^{###}P< 0.001$; $*P < 0.05$, $**P < 0.01$, $***P < 0.001$; Values are presented as mean ± SEM, n=6. Statistical analysis was performed by two-tailed Student's t test.

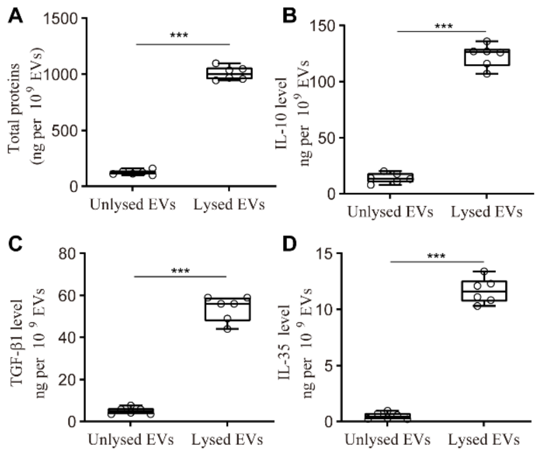

Figure S5. The quantification of protein in lysed and unlysed Foe-TEVs. (A) The total proteins detected by BCA assay in lysed and unlysed Foe-TEVs at 10^9 particles. (B-D) The protein level of IL-10, TGF-β1 and IL-35 in lysed and unlysed Foe-TEVs at 10^9 particles. ***P < 0.001; Values are presented as mean ± SEM, n=6. Statistical analysis was performed by two-tailed Student's t test.

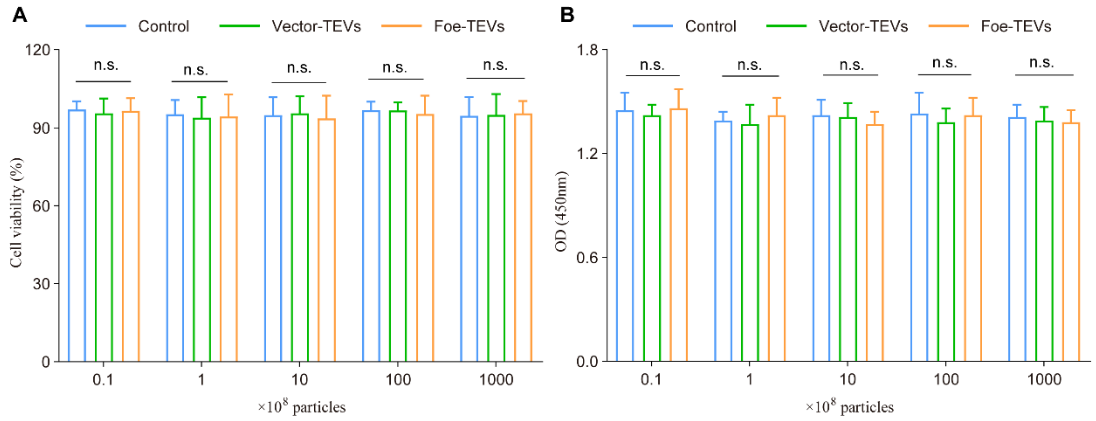

Figure S6. Survival of HUVEC in control, Vector-TEVs and Foe-TEVs groups under gradient TEVs particles. (A) Live/Dead staining assay. (B) CCK-8 cell proliferation assay. HUVECs, human umbilical vein endothelial cells; n.s., not significant; Values are presented as mean ± SEM, n=6. Statistical analysis was performed by the one-way analysis of variance.

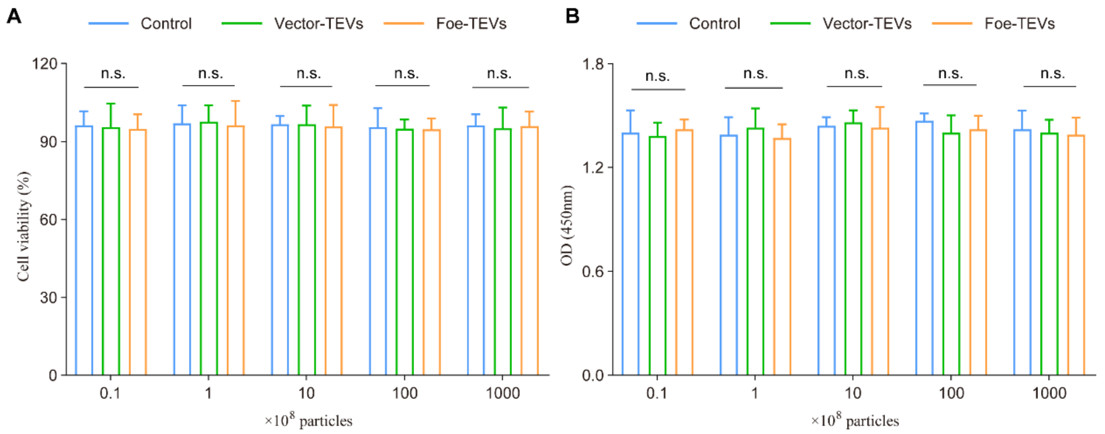

Figure S7. Survival of KFB in control, Vector-TEVs and Foe-TEVs groups under gradient TEVs particles. (A) Live/Dead staining assay. (B) CCK-8 cell proliferation assay. KFBs, kidney fibroblasts; n.s., not significant; Values are presented as mean ± SEM, n=6. Statistical analysis was performed by the one-way analysis of variance.

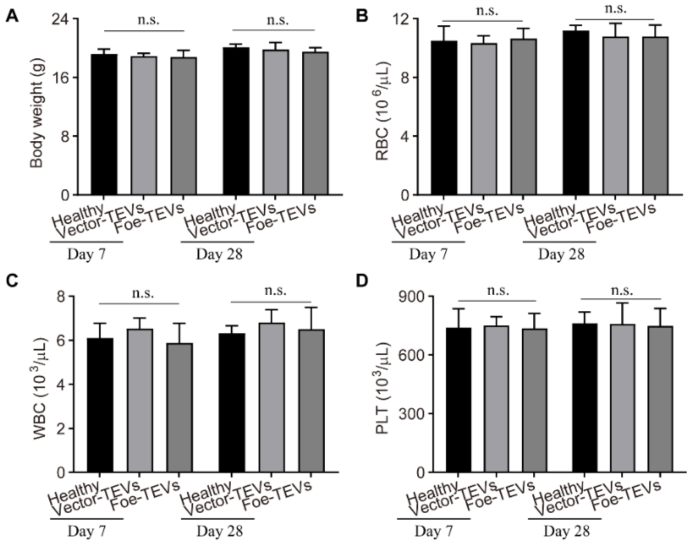

Figure S8. Body weight and haematological parameters upon administration of Foe-TEVs. (A) Mouse Body weights on day 7 and day 28 after healthy mice injected with Vector-TEVs, Foe-TEVs or without any injection. (B) RBC count in whole blood of mice on day 7 and day 28 after healthy mice injected with Vector-TEVs, Foe-TEVs or without any injection. (C) WBC count in whole blood of mice on day 7 and day 28 in each group. (D) PLT count in whole blood of mice on day 7 and day 28 in each group. RBC, red blood cell; WBC, white blood cell; PLT, platelet; n.s., not significant; Values are presented as mean ± SEM, n=6. Statistical analysis was performed by the one-way analysis of variance.

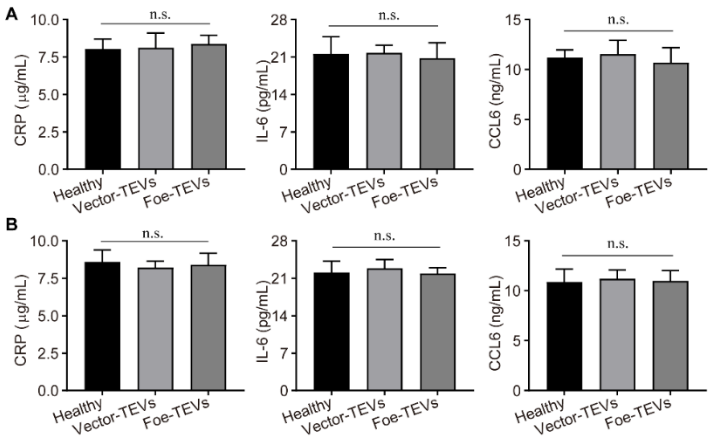

Figure S9. Blood level of inflammatory cytokines upon administration of Foe-TEVs in vivo. (A) Representative blood level of inflammatory cytokines (CRP, IL-6 and CCL6) on day 7 after healthy mice injected with Vector-TEVs, Foe-TEVs or without any injection. (B) Representative blood level of inflammatory cytokines (CRP, IL-6 and CCL6) on day 28 after healthy mice injected with Vector-TEVs, Foe-TEVs or without any injection. CRP, C-Reactive Protein; IL-6, interleukin-6; CCL6, Chemokine (C-C motif) ligand 6; n.s., not significant; Values are presented as mean ± SEM, n=6. Statistical analysis was performed by the one-way analysis of variance.

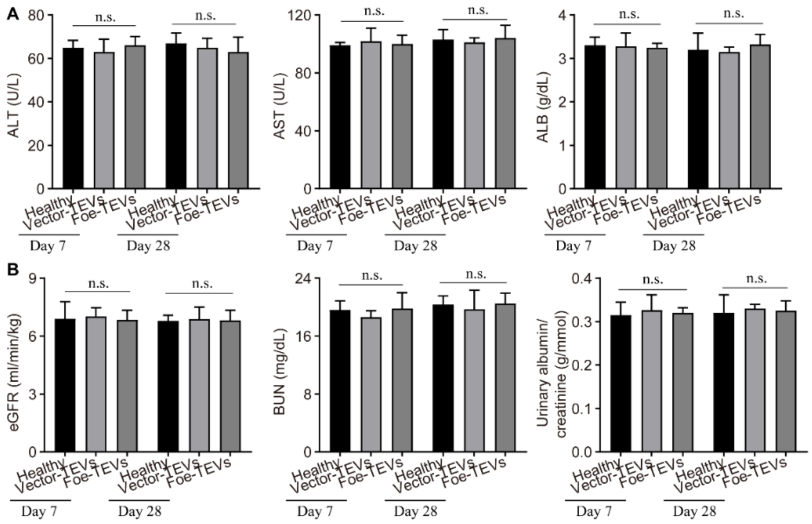

Figure S10. Blood level of liver function and kidney function upon administration of Foe-TEVs in vivo. (A) Blood level of liver-damage markers (AST, ALT, and ALB) on day 7 and day 28 after healthy mice injected with Vector-TEVs, Foe-TEVs or without any injection. (B) Blood level of kidney-damage markers (eGFR, BUN and urinary protein/creatinine ratio) on day 7 and day 28 after healthy mice injected with Vector-TEVs, Foe-TEVs or without any injection. AST, aspartate transaminase; ALT, alanine transaminase; ALB, albumin; eGFR, estimated glomerular filtration rate; BUN, blood urea nitrogen; n.s., not significant; Values are presented as mean ± SEM, n=6. Statistical analysis was performed by the one-way analysis of variance.

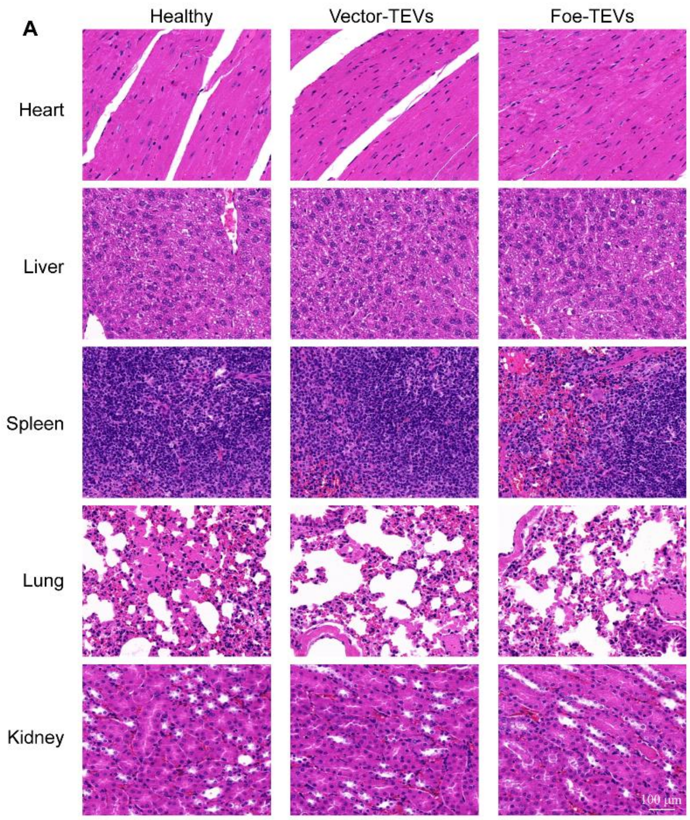

Figure S11. Biocompatibility of Foe-TEVs in vivo. (A) H&E staining results of heart, liver, spleen, lung and kidney of healthy mice or healthy mice treated with Vector-TEVs or Foe-TEVs.

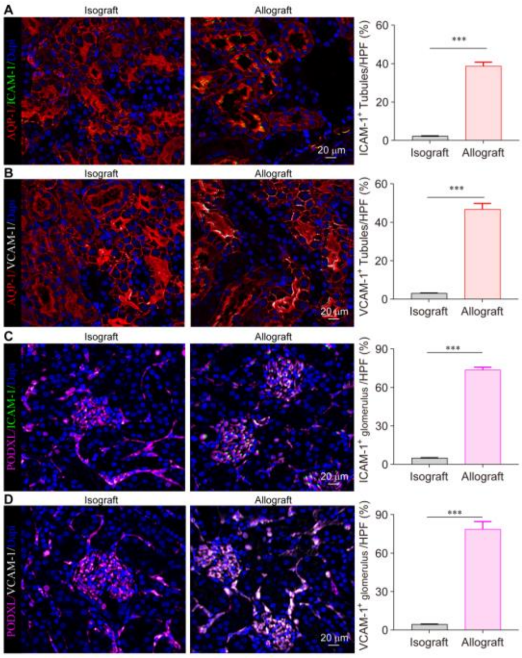

Figure S12. Expression level of ICAM-1 and VCAM-1 in renal tubular and glomerular sites. (A and C) Representative immunofluorescence images showing ICAM-1 staining level in the renal tubular and glomerular sites of isograft and allograft groups. (B and D) Representative immunofluorescence images showing VCAM-1 staining level in the renal tubular and glomerular sites of isograft and allograft groups. ***P<0.001. Values are presented as mean ± SEM, n=6. Statistical analysis was determined by two-tailed Student's test.

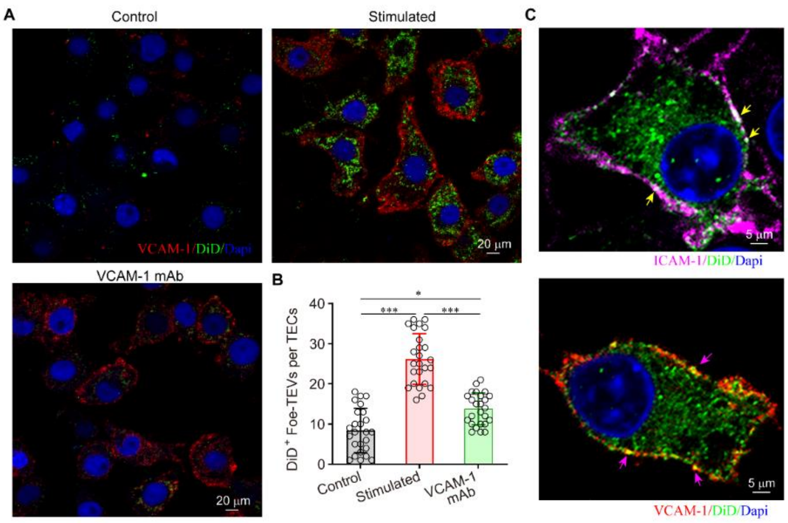

Figure S13. Representative images of DiD-labeled Foe-TEVs adhere to VCAM-1 on HK-2 cells stimulated with IL-1β and IFN-γ. (A) Confocal imaging of DiD-labeled Foe-TEVs adhere to VCAM-1 on HK-2 cells in control group, stimulated group and VCAM-1 monoclonal antibody blocking group. (B) Statistical data on DiD^+ area (n=25). (C) STED microscopy capturing the interaction and adhesion between DiD-labeled Foe-TEVs and ICAM-1/VCAM-1. Yellow arrow presented co-localization and interaction between DiD-labeled Foe-TEVs and ICAM-1 on HK-2 cells. Magenta arrow presented co-localization and interaction between DiD-labeled Foe-TEVs and VCAM-1 on HK-2 cells. *P<0.05, ***P < 0.001; Values are presented as mean ± SEM. Statistical analysis was performed by two-tailed Student's t test.

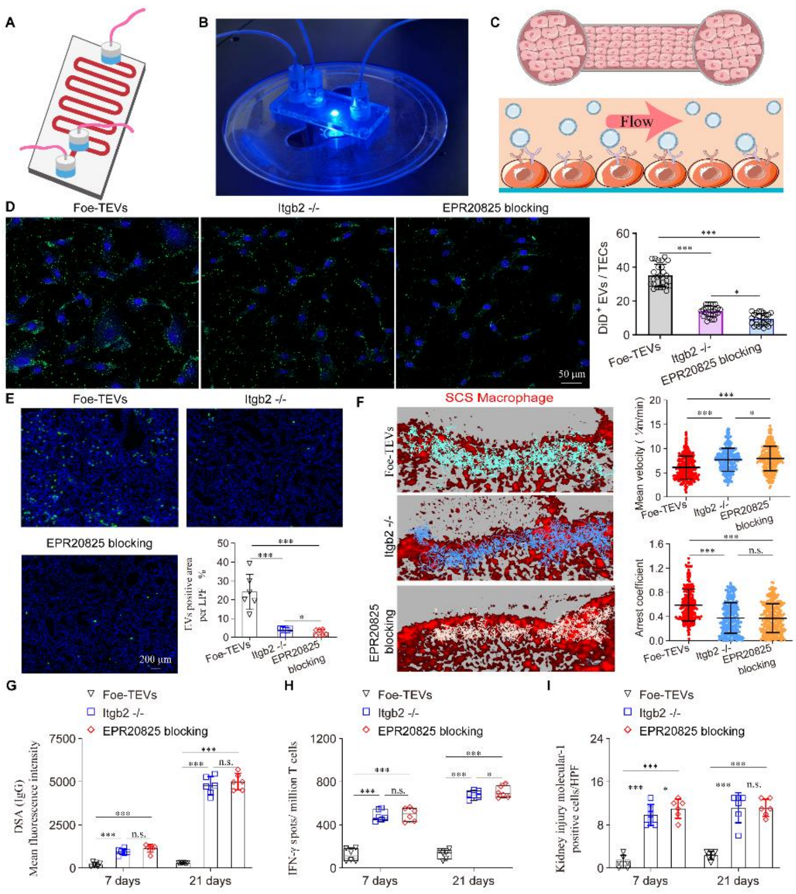

Figure S14. Itgb2 knockout or integrins blocking suppressed Foe-TEVs recruitment and adhesion to allograft and secondary lymphoid organs. (A to C) The schematic illustration of microfluidic chip used to simulate the process of Foe-TEVs adhesion to HK-2 cells. (D) Confocal imaging showing the DiD-labeled Foe-TEVs positive in activated HK-2 cells in Foe-TEVs group, Itgb2$^{-/-}$ Foe-TEVs group, and EPR20825 blocking group (n=25). (E) The distribution of Foe-TEVs, Itgb2$^{-/-}$ Foe-TEVs and EPR20825 blocking Foe-TEVs in allografts detected by immunofluorescence (n=6). (F) Confocal images showing tracks, the mean velocity and the arrest coefficient of Foe-TEVs, Itgb2$^{-/-}$ Foe-TEVs and EPR20825 blocking Foe-TEVs contacting with SCS macrophages under subcapsular sinus of lymph nodes (n=300). (G) MFI detection of total DSA levels in serum at 7 and 21 days posttransplant (n=6). (H) Kinetics of alloreactive T cell responses at 7 and 21 days posttransplant (n=6). (I) Cell counting of kidney injury molecular positive in high-power field (HPF) at 7 days and 21 days posttransplant (n=6). SCS, subcapsular sinus; DSA, donor specific antibody; n.s., not significant; *P<0.05, ***P < 0.001; Values are presented as mean ± SEM.

Statistical analysis was performed by two-tailed Student’s t test.

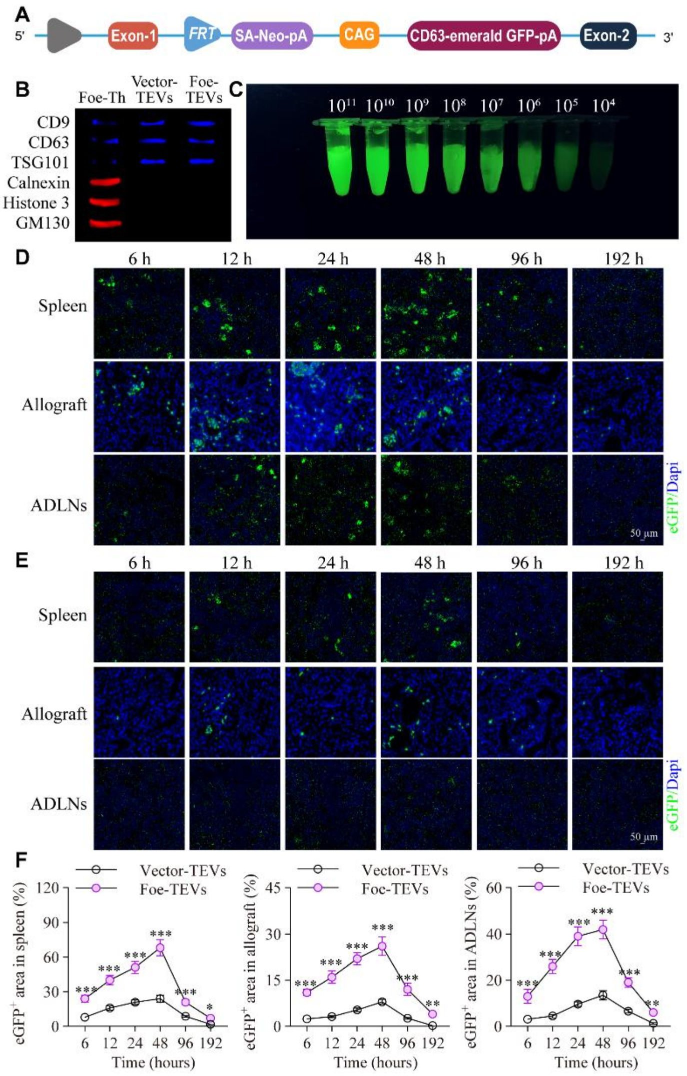

Figure S15. The in vivo biodistribution of bioluminescent labelling EVs. (A) Scheme showing the bioluminescent labelling of EVs using green fluorescent protein fused to CD63 sequence. (B) Western blot analysis of CD9, CD63, TSG101, Calnexin, Histone 3 and GM130 expression in bioluminescent labelling Foe-Th cells, Vector-TEVs and Foe-TEVs. (C) The bioluminescence of EVs with different concentrations, under the 488 nm excitation light. (D) The distribution of Foe-TEVs in spleen, renal allograft and ADLNs detected by immunofluorescence. (E) The distribution of Vector-TEVs in spleen, renal allograft and ADLNs detected by immunofluorescence. (F) Quantitative emGFP positive area in spleen, ADLNs and renal allograft tissues at different time series (n=6). GFP, green fluorescent protein; ADLNs, allograft draining lymph nodes; $*P<0.05$, $**P<0.01$, $***P<0.001$; n.s., not significant compared with the Vector-TEVs

group; Values are showed as mean ± SEM. Statistical analysis was determined by two-tailed Student's t test.

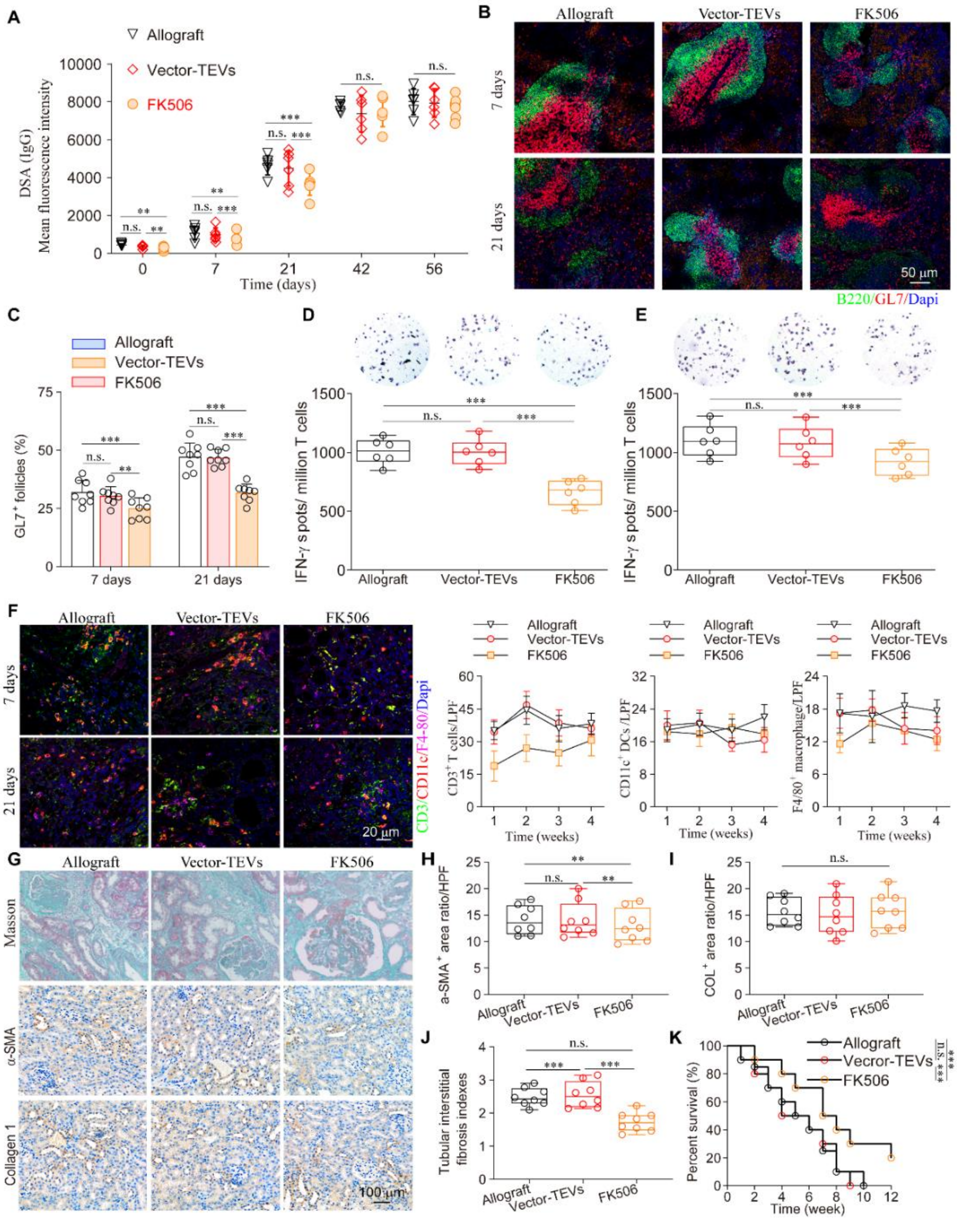

Figure S16. Allosensitization kinetics, renal fibrosis and the survival period of recipients after allograft transplant without any treatment. (A) MFI detection of total DSA levels in serum at day 0, day 7, day 21, day 42 and day 56 posttransplant in the Allograft, Vector-TEVs and FK506 groups (n=6). (B) Immunofluorescence staining of GL7$^+$ GC B cells located within the follicle (B220$^+$) in spleen at 7 days and 21 days posttransplant, respectively. (C) The quantitative statistics of GL7$^+$ follicles (n=8). (D) Kinetics of alloreactive T cell responses at 7 days posttransplant (n=6). (E) Kinetics of alloreactive T cell responses at 21 days posttransplant (n=6). (F) Representative immunofluorescence staining of F4/80$^+$ macrophages, CD3$^+$ T cells as well as CD11c$^+$

DCs in the graft at 7 days and 21 days posttransplant. Quantification of the infiltration of T cells, DCs and macrophages in the graft (n=8). *P<0.05, compared to the Vector-TEVs group; #P< 0.05, compared with the FK506. (G) Masson staining and representative immunohistochemical evaluation of a-SMA and collagen I after 8th week postoperative. (H-I) The analysis of α-SMA and type I Collagen positive area ratio in each group after 8th week postoperative (n=8). (J) Fibrotic index of renal interstitial fibrosis at 8 weeks post-operation (n=8). (K) Survival percentage of allograft recipients with different treatments (n=8). DSA, donor specific antibody; GC, germinal center; n.s., not significant; *P<0.05, **P<0.01, ***P<0.001, #P< 0.05, ##P< 0.01, ###P< 0.001. Values are presented as mean ± SEM. Statistical analysis was determined by two-tailed Student's t test.

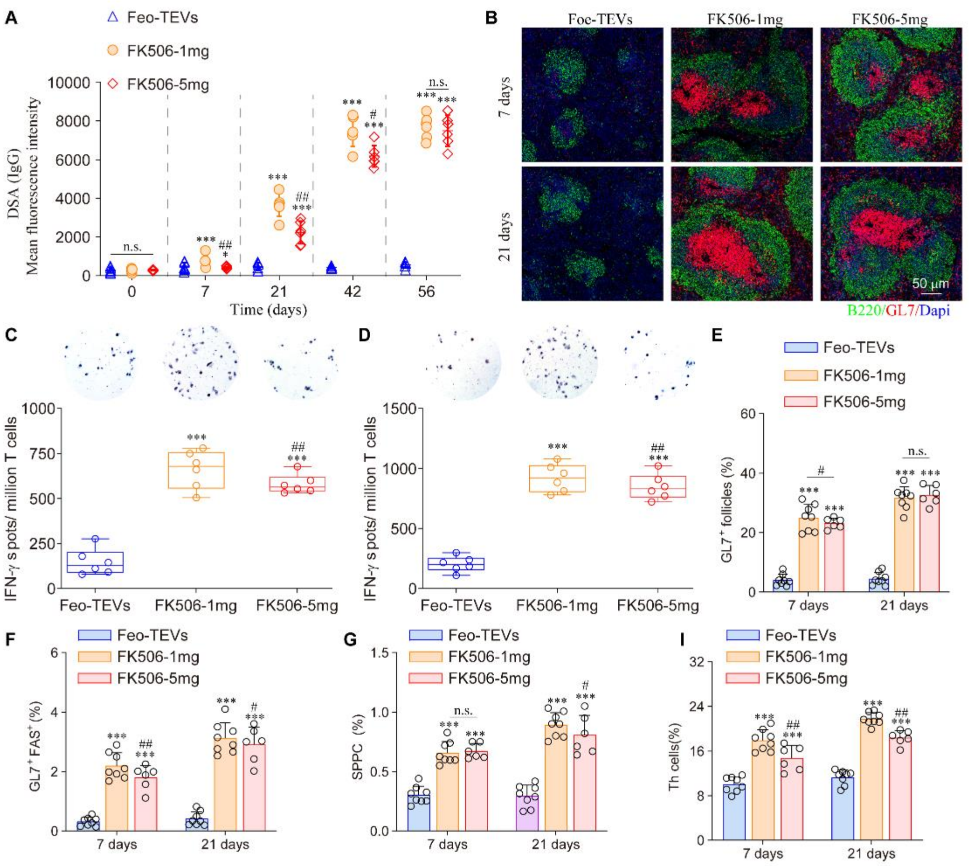

Figure S17. Allosensitization kinetics of mice treated with Foe-TEVs or FK506. (A) MFI detection of total DSA levels in serum at day 0, day 7, day 21, day 42 and day 56 posttransplant, respectively (n=6). (B) Immunofluorescence staining of GL7^{+} GC B cells located within the follicle (B220^{+}) in spleen at 7 days and 21 days posttransplant, respectively. (C) Kinetics of allospecific T cell responses at 7 days posttransplant (n=6). (D) Kinetics of allospecific T cell responses at 21 days posttransplant (n=6). (E) The quantitative statistics of GL7^{+} germinal centers in (B) at 7 days and 21 days posttransplant (n=8). (F-I) The quantitative statistics of GL7^{+} FAS^{+} GC B cells (F), CD138^{+} SPPCs (G) and CD4^{+} Th cells (I) at day 7 and day 21 posttransplant respectively (n=8). DSA, donor specific antibody; GC, germinal center; SPPC, splenic plasma cell; n.s., not significant; *<0.05, ***P<0.001 compared with the Foe-TEVs group; #P <0.05, ##P< 0.01 compared with the FK506-1mg group. Values are presented as mean ± SEM. Statistical analysis was determined by two-tailed Student's t test.

Table S1. Primers used for qRT-PCR.

Abbreviation	Sequences	
	Forward	Reverse
FOXP3	5’-CCTGGTTGTGAGAAGGTC TTCG-3’	5’-TGCTCCAGAGACTGCACC ACTT-3’
GAPDH	5’−CATCACTGCCACCCAGAA GACTG -3’	5’- ATGCCAGTGAGCTTCCCGT TCAG -3’
